# Evaluating the Validity and Utility of Wearable Technology for Continuously Monitoring Patients in a Hospital Setting: Systematic Review

**DOI:** 10.2196/17411

**Published:** 2021-08-18

**Authors:** Vikas Patel, Ani Orchanian-Cheff, Robert Wu

**Affiliations:** 1 Faculty of Medicine University of Toronto Toronto, ON Canada; 2 Library and Information Services University Health Network Toronto, ON Canada; 3 Division of General Internal Medicine University Health Network Toronto, ON Canada

**Keywords:** wearable, inpatient, continuous monitoring

## Abstract

**Background:**

The term *posthospital syndrome* has been used to describe the condition in which older patients are transiently frail after hospitalization and have a high chance of readmission. Since low activity and poor sleep during hospital stay may contribute to posthospital syndrome, the continuous monitoring of such parameters by using affordable wearables may help to reduce the prevalence of this syndrome. Although there have been systematic reviews of wearables for physical activity monitoring in hospital settings, there are limited data on the use of wearables for measuring other health variables in hospitalized patients.

**Objective:**

This systematic review aimed to evaluate the validity and utility of wearable devices for monitoring hospitalized patients.

**Methods:**

This review involved a comprehensive search of 7 databases and included articles that met the following criteria: inpatients must be aged >18 years, the wearable devices studied in the articles must be used to continuously monitor patients, and wearables should monitor biomarkers other than solely physical activity (ie, heart rate, respiratory rate, blood pressure, etc). Only English-language studies were included. From each study, we extracted basic demographic information along with the characteristics of the intervention. We assessed the risk of bias for studies that validated their wearable readings by using a modification of the Consensus-Based Standards for the Selection of Health Status Measurement Instruments.

**Results:**

Of the 2012 articles that were screened, 14 studies met the selection criteria. All included articles were observational in design. In total, 9 different commercial wearables for various body locations were examined in this review. The devices collectively measured 7 different health parameters across all studies (heart rate, sleep duration, respiratory rate, oxygen saturation, skin temperature, blood pressure, and fall risk). Only 6 studies validated their results against a reference device or standard. There was a considerable risk of bias in these studies due to the low number of patients in most of the studies (4/6, 67%). Many studies that validated their results found that certain variables were inaccurate and had wide limits of agreement. Heart rate and sleep were the parameters with the most evidence for being valid for in-hospital monitoring. Overall, the mean patient completion rate across all 14 studies was >90%.

**Conclusions:**

The included studies suggested that wearable devices show promise for monitoring the heart rate and sleep of patients in hospitals. Many devices were not validated in inpatient settings, and the readings from most of the devices that were validated in such settings had wide limits of agreement when compared to gold standards. Even some medical-grade devices were found to perform poorly in inpatient settings. Further research is needed to determine the accuracy of hospitalized patients’ digital biomarker readings and eventually determine whether these wearable devices improve health outcomes.

## Introduction

### Background

Most physiologic parameters, such as vital signs or activity, are routinely monitored a few times each day in hospital ward settings [1]. Some parameters, such as sleep, are not routinely monitored at all [2,3]. More frequent monitoring could allow for the timely identification of the deteriorating health of patients and spur efforts for improving patients’ overall health through increased sleep and activity. Since subtle changes in vital signs are often present 8 to 24 hours before a life-threatening event, such as intensive care unit admission or cardiac arrest, vital sign surveillance has the potential to detect clinical deterioration at an earlier phase, thereby permitting clinicians to make corrective interventions [4-7]. This includes identifying patients with poorly controlled pain and recognizing arrhythmias. The term *posthospital syndrome* has been used to denote the deleterious effects of acute illnesses that are compounded with poor sleep and low activity and occur during hospital stay [8]. Measuring sleep and activity could improve the recognition of such issues and encourage health providers to introduce interventions that improve patients’ experiences in hospitals by encouraging mobilization and to identify targets for sleep-promoting interventions [9-11]. In addition, access to other digital biomarkers (eg, heart rate, blood pressure, oxygen saturation, etc) would allow clinicians to determine underlying etiologies and make tailored interventions.

The rapid uptake of affordable wearables, such as fitness bands, may provide a method for continuously measuring sleep; activity; and vital signs, such as heart rate [12-15]. However, existing literature that describes wearable devices is mostly limited to ambulatory settings and focuses on the management of chronic diseases [16,17]. More inpatient data are needed on both the validity of wearables and patient adherence. Although wearable testing has been conducted with healthy volunteers, it will be important to validate these signals in inpatient settings, where algorithms for processing sensor data into digital signals, such as those for sleep, heart rate, and activity, may be less accurate [18]. Despite the proposed benefit of intensive monitoring, many wearable studies have found issues with patient adherence [18-20]. Adherence is a crucial barrier to acquiring data and can be influenced by device convenience, the comfort of use, and interaction requirements [19]. Studies of wearable devices worn by hospitalized inpatients have been limited by large dropout rates [20].

Although there have been systematic reviews of the monitoring of patients’ physical activity in hospitals [21-23], there are no reviews of the use of wearables that can reliably measure other health parameters. Therefore, in this review, we aimed to expand our search by including articles that used wearables to assess parameters other than physical activity and to assess the adherence of patients in inpatient settings.

### Objective

For the purposes of this review, a wearable was considered to be any electronic device that has at least 1 sensor and can be worn on the body [24]. Wearables were examined for their ability to measure digital biomarkers, which are defined as digitally collected physiological and behavioral measures (eg, heart rate, average sleep duration, and daily step count) that explain, influence, or predict health-related outcomes [18]. Consistent with previous research, patient adherence was objectively assessed by reporting the mean proportion of patients who completed a given study [25]. The primary objectives of this review were to determine patients’ adherence to using wearable devices in hospitals and to examine the validity of wearable-derived biomarker readings.

## Methods

### Identification and Selection of Studies

A comprehensive search strategy was developed to identify articles on the three main concepts of our question—wearables, monitoring, and inpatients. The initial search strategy was developed for Ovid MEDLINE by using a combination of database-specific subject headings and text words (Multimedia Appendix 1). Additional key words were generated based on input from the subject specialists on the team, and the revised search strategy was customized for each database.

Searches of the following databases were executed on August 16, 2018: Ovid MEDLINE, Ovid MEDLINE Epub Ahead of Print and In-Process & Other Non-Indexed Citations, Cochrane Database of Systematic Reviews, Cochrane Central Register of Controlled Trials, Health Technology Assessment database (Ovid), and CINAHL with Full Text. The search in Ovid Embase was not executed until September 5, 2018, due to issues with the vendor’s August database reload. Additional search methods included reviewing the cited references of eligible studies via Web of Science (May 6, 2019) and the reference lists of eligible studies. There were no restrictions on publication period. Limits were imposed to ensure that only English-language studies and those with adult populations were included in this review. No other limits were applied to the literature search.

### Article Selection and Exclusion Criteria

Records were screened by two reviewers (VP and RW) independently. For selected studies, full-text articles were obtained and evaluated for eligibility [26]. The eligibility criteria for inclusion in this review were as follows:

- Medical or surgical inpatients aged >18 years
- Device studied in the article must be a wearable (such as a watch, vest, pendant, jewelry, headset, and wristband)
- Articles must describe an element of continuous monitoring for at least 24 hours or greater
- Articles must describe the measurement of 1 or more digital biomarkers other than just physical activity or standard hospital telemetry for heart rate recording.

We excluded articles that were not considered original research, such as letters to the editor, comments, and reviews. We also excluded articles that monitored less than 3 patients, described the monitoring of a very specialized system in the body (eg, insole devices, ventricular assistive devices, and cochlear implants), involved the monitoring of patients in rehabilitation hospitals, or used wearables as tools for therapy (eg, insulin delivery).

### Data Extraction

Two reviewers (RW and VP) independently extracted the data and resolved any disagreements by discussing the findings and making a collective decision. The data extracted for each article included the year of publication, study setting and design, number of participants, gender ratio, mean age of participants, digital biomarkers measured in the study, average and maximum duration that the wearable was worn by participants in each study, and patient completion rate (the proportion of patients that wore the wearable for the minimum monitoring duration that was set by the study authors). For studies that used a reference standard, any participants who were missing data from the wearable or the standard were determined to be incomplete measurement pairs and were omitted from the final count of patients who completed the study. Furthermore, we extracted the types of wearables that were worn by the participants in each study along with the placement sites on the body. Devices were classified as medical grade (approved or cleared by the US Food and Drug Administration), research grade (typically used in research settings only), and consumer grade (used by general consumers).

Validation data were also collected for each article by assessing whether the authors compared the accuracy of their digital readings to a reference standard. To determine the validity of measures that were compared to a reference standard, correlation coefficients, mean differences, and limits of agreement were extracted from each study.

### Risk of Bias Assessment

All articles that assessed for validated readings were independently assessed for their risk of bias by two independent reviewers (VP and RW) using a modification of the validation subscale from a checklist for assessing the methodological quality of studies on the measurement properties of health status measurement instruments (Consensus-Based Standards for the Selection of Health Status Measurement Instruments [COSMIN]) [27] (Table 1). All discrepancies were resolved by discussion and consensus. The quality evaluation included 5 study design and methodology components (the percentage of missing data, missing data management, adequate sample size, acceptable criterion comparison, and design or methodological flaws) and 1 analysis component (acceptable accuracy analyses). We rated the quality of each dimension as excellent, good, fair, or poor based on a priori modifications to the COSMIN validation subscale for scoring criteria that are appropriate for accuracy studies (Multimedia Appendix 2) [28].

**Table 1:** Risk of bias assessment for studies that validated their wearable readings.

Study	Assessment criterion									
	Mean or % difference	Correlation	LOA^a^	Percentage of missing data	Missing data management	Adequate sample size (patients)	Adequate sample size (measurements)	Acceptable reference comparison	Other methodological flaws	Acceptable accuracy analyses
Bloch et al [29]	No	No	No	Excellent	Excellent	Poor	Poor	Excellent	No	Poor
Breteler et al [30]	Yes	No	Yes	Excellent	Excellent	Poor	Excellent	Excellent	No	Excellent
Gallo and Lee [13]	No	Yes	No	Excellent	Excellent	Fair	Fair	Fair	No	Excellent
Kroll et al [11,31]	Yes	Yes	Yes	Excellent	Excellent	Good	Excellent	Excellent	No	Excellent
Steinhubl et al [32]	No	Yes	No	Excellent	Excellent	Poor	Excellent	Excellent	No	Excellent
Weenk et al [4]	Yes	No	Yes	Excellent	Excellent	Poor	Good	Excellent	No	Excellent

^a^LOA: limits of agreement.

## Results

### Characteristics of Included Studies

**Figure 1:**
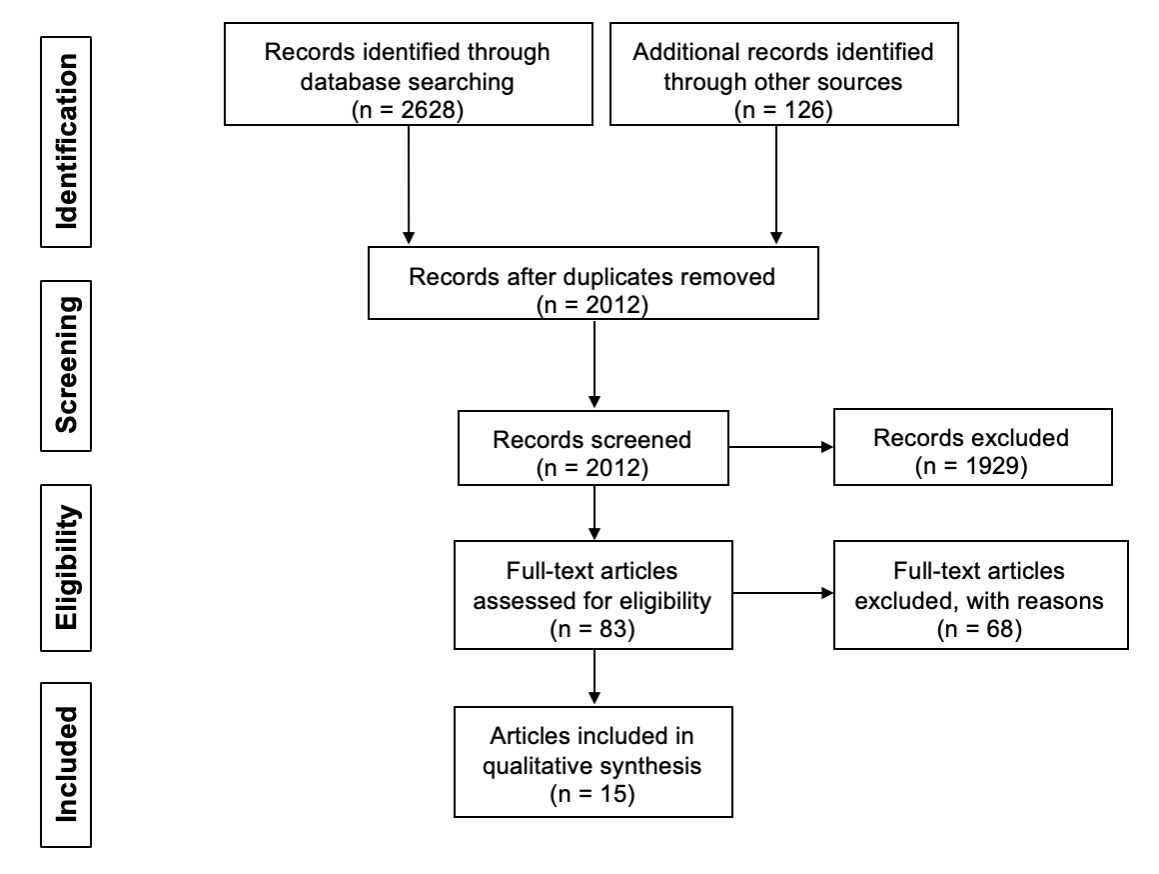
PRISMA (Preferred Reporting Items for Systematic Reviews and Meta-Analyses) flow diagram of included and excluded studies.

Our literature search identified 2754 article citations. After excluding duplicate records, 2012 records were deemed eligible for screening. A total of 83 studies were selected based on abstracts and underwent full-text review. After applying our inclusion and exclusion criteria, 15 articles that described 14 studies were selected for this review (Figure 1).

**Figure 2:**
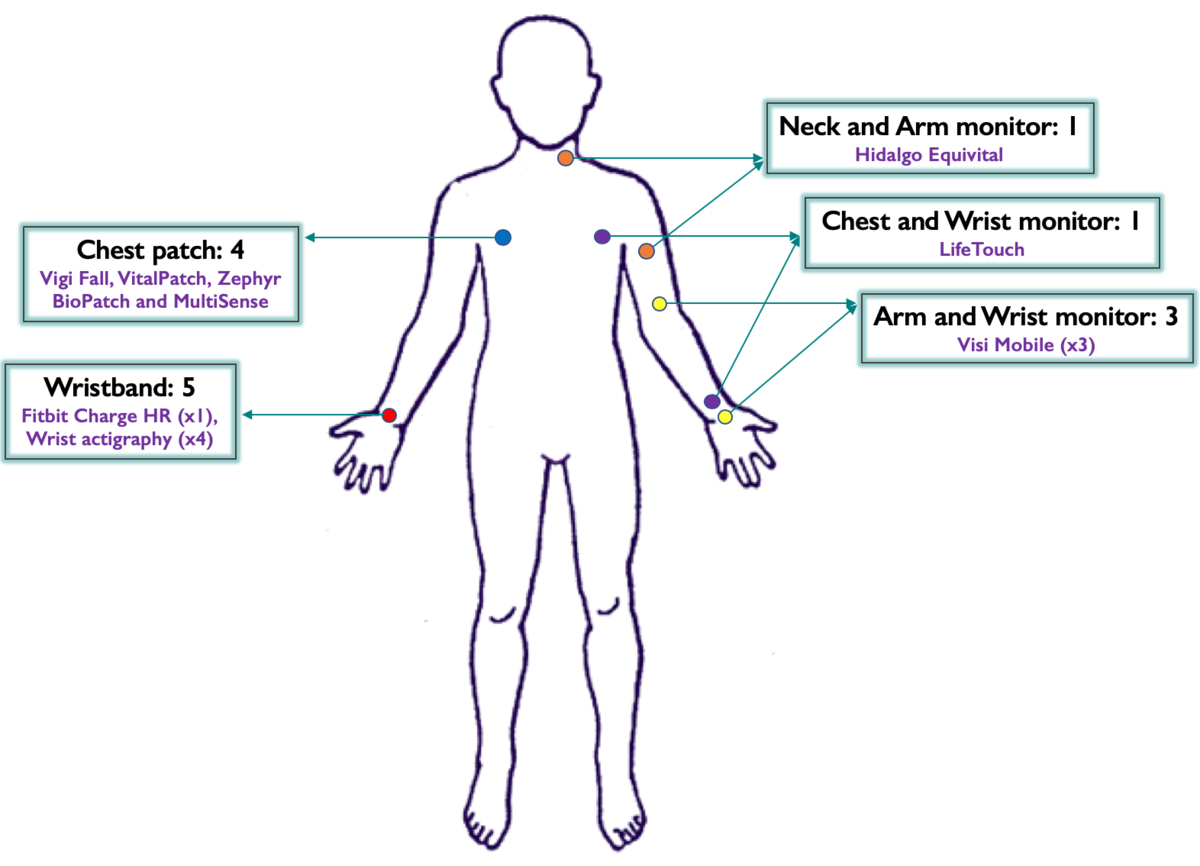
Illustration of the types of and body locations for used wearable devices.

All of the articles included were prospective cohort studies (Table 2) [4,11,13,14,20,29-38]. Overall, 9 different types of commercial wearables were described across the 14 studies, and 7 different health variables were assessed collectively by the 14 studies (Table 3). The wearable devices that were described by the studies came in various different forms and were attached to a range of sites on the body (Figure 2). A total of 13 articles included both men and women as the study participants; the other two papers assessed sleep changes in postpartum women [13,34]. Kroll et al [11,31] published two articles from the same study. Both articles analyzed different aspects of the continuous monitoring of inpatients (ie, they used the same cohort of patients) but were included as the same study entry in this review (Table 2).

Collectively, the mean patient completion rate across all 14 studies was over 90%. Of the 8 articles that included a qualitative analysis as a part of their methodology, 7 reported that wearables were well received by either or both patients and clinicians.

Of the 14 studies, 6 validated wearable measurements against another standard device or measure (Table 3). The studies conducted by Bloch et al [29], Gallo and Lee [13], and Steinhubl et al [32] used intermittent measurements (nurse or questionnaires) for their reference standard. Further, Breteler et al [30] used a continuous reference (continuous electrocardiography and impedance pneumography) to compare the wearable readings for heart rates and respiratory rates [30]. Weenk et al [4] and Kroll et al [11,31] validated their wearable readings against both intermittent and continuous reference measurements. Of the 9 wearables included in the studies, 6 were cleared or approved by the US Food and Drug Administration as medical devices (ViSi Mobile [Sotera Wireless], Hidalgo EQ02 [Equivital], wrist actigraphy [Ambulatory Monitoring Inc; Actigraph LLC], LifeTouch [Isansys Lifecare], Zephyr Biopatch [Medtronic], and HealthPatch [VitalConnect]).

**Table 2:** Summary of included studies.

Study	Year published	Setting (ward)	Methodology	Patients, N	Male %: Female % ratio, mean age (years)	Variables measured	Number of days device was worn, average (maximum)	Patient completion rate, %
Lee and Lee [34]	2007	Obstetric	Prospective cohort	21	Females only, 32	Sleep	2^a^	100
Gallo and Lee [13]	2008	Obstetric	Prospective cohort	39	Females only, 29	Sleep	2 (2)	100
Bloch et al [29]	2011	Geriatric	Prospective cohort	10	Males and females^b^, 83	Falls	21^a^	90
Chiu et al [33]	2013	Neurosurgery	Prospective cohort	60	65:35, 35	Sleep	7^a^	87
Watkins et al [37]	2015	Medicine and surgical	Prospective cohort	236	Males and females^b,c^	HR^d^, RR^e^, SpO_2_^f^, and BP^g^	3 (3)	100
Jeffs et al [20]	2016	Medicine	Prospective cohort	208	72:28^c^	HR. RR, SpO_2_, temperature, and accelerometry	(14)^h^	32
Steinhubl et al [32]	2016	Medicine	Prospective cohort	26	65:35, 33	HR, RR, and temperature	3 (3)	100
Razjouyan et al [35]	2017	Hematology and oncology	Prospective cohort	35	45:55, 55	HR and fall risk	1^a^	94
Weenk et al [4]	2017	General internal medicine and surgical	Prospective cohort	20	65:35, 50	HR, RR, BP, SpO_2_, and temperature	2.5 (3)	100
Kroll et al [11,31]	2017	Intensive care unit	Prospective cohort	50	52:48, 64	HR, sleep	1^a^	96
Weller et al [36]	2017	Neurology and neurosurgery	Prospective cohort	736	54:46^c^	HR, RR, SpO_2_, and BP	1.7 (9)	100
Breteler et al [30]	2018	Surgical	Prospective cohort	33	72:28, 63	HR and RR	2.6 (3)	76
Yang et al [14]	2018	Oncology	Prospective cohort	11	64:36^c^	Sleep	16^a^	91
Duus et al [38]	2018	General surgery	Prospective cohort	50	58:42, 71	HR, RR, and SpO_2_	3.1 (4)	100

^a^The maximum number of days was not reported in the study.

^b^The study included both male and female participants but did not report a ratio.

^c^Mean age was not reported in the study.

^d^HR: heart rate.

^e^RR: respiratory rate.

^f^SpO_2_: oxygen saturation

^g^BP: blood pressure.

^h^The average number of days was not reported in the study.

**Table 3:** Distribution of the health variables that were assessed for accuracy in each study.

Study	Device characteristics		Digital biomarkers						
	Device, manufacturer	FDA^a^ clearance or approval	Heart rate	Sleep	Respiratory rate	SpO_2_^b^	Skin temperature	Blood pressure	Fall risk
Gallo and Lee [13]	Wrist Actigraph, Ambulatory Monitoring Inc	Yes	—^c^	*R*=0.53	—	—	—	—	—
Lee and Lee [34]	Mini-MotionloggerActigraphy, Ambulatory Monitoring Inc	Yes	—	Not validated	—	—	—	—	—
Chiu et al [33]	ActiGraph GT1M, Actigraph LLC	Yes	—	Not validated	—	—	—	—	—
Yang et al [14]	Actigraph GT3X+ watch, Actigraph LLC	Yes	—	Not validated	—	—	—	—	—
Kroll et al [11,31]	Fitbit Charge HR, Fitbit Inc	—	LoA^d^ (sinus): 23.9 to 21.9 beats per minute	*R*=0.33	—	—	—	—	—
Breteler et al [30]	HealthPatch, VitalConnect	Yes	LoA: −8.8 to 6.5 beats per minute	—	LoA: −15.8 to 11.2 breaths per minute	—	Not validated	—	Not validated
Jeffs et al [20]	Hidalgo EQ02, Equivital	Yes	Not validated	—	Not validated	Not validated	Not validated	—	—
Duus et al [38]	LifeTouch, Isansys Lifecare	Yes	Not validated	—	Not validated	Not validated	—	—	—
Steinhubl et al [32]^e^	MultiSense patch, Rhythm Diagnostic Systems	—	*R*=0.75	—	*R*=0.83	—	*R*=0.99	—	—
Bloch et al [29]	Vigi’Fall, Vigilio Telemedical	—	—	—	—	—	—	—	Sensitivity: 37.5%
Weenk et al [4]	ViSi Mobile, Sotera Wireless	Yes	LoA: −11.1 to 10.7 beats per minute	—	−5.5 to 7.9 breaths per minute	−3.1% to 3.3%	Not validated	SBP^f^: −23 to 24 mm Hg; DBP^g^: 27.5 to 11.5 mm Hg	—
Weenk et al [4]	HealthPatch, VitalConnect	Yes	−12.6 to 9.5 beats per minute	—	−10.3 to 9.0 breaths per minute	Not validated	Not validated	Not validated	—
Weller et al [36]	ViSi Mobile, Sotera Wireless	Yes	Not validated	—	Not validated	Not validated	Not validated	Not validated	—
Watkins et al [37]	ViSi Mobile, Sotera Wireless	Yes	Not validated	—	Not validated	Not validated	—	Not validated	—
Razjouyan et al [35]	Zephyr BioPatch, Medtronic	Yes	Not validated	—	—	—	—	—	Not validated

^a^FDA: US Food and Drug Administration.

^b^SpO_2_: oxygen saturation.

^c^Not available.

^d^LOA: limits of agreement.

^e^Steinhubl et al [32] did not report limits of agreement.

^f^SBP: systolic blood pressure.

^g^DBP: diastolic blood pressure.

### Risk of Bias

Of the 6 studies in the risk of bias assessment, 4 were ranked as poor due to a small sample size (participants: N<30). The study conducted by Gallo and Lee [13] used sleep questionnaires as a reference measure and therefore received a fair rating for the “acceptability of reference” criterion, whereas the other five studies were ranked as excellent (ie, they used intermittent nurse readings or other validated methodologies). Further, in terms of assessing the accuracy analyses, only the study conducted by Bloch et al [29] did not report mean differences, correlations, and limits of agreement.

### Validation by Digital Biomarker

#### Heart Rate

A total of 5 studies assessed heart rate accuracy. Breteler et al [30] found that the bias and 95% limits of agreement for heart rate were −1.1 beats per minute (BPM) and −8.8 to 6.5 BPM, respectively, for 55,565 heart rate pairs [30]. Specifically, the wearable sensor accurately detected tachycardia with a sensitivity of 90% and a specificity of 97% [30]. In a cohort of intensive care unit patients, Kroll et al [11,31] found that the Fitbit (Fitbit Inc)-derived heart rate values were slightly lower than those derived from continuous electrocardiography monitoring but that 73% of the readings were within 5 BPM of the electrocardiogram value (average bias: −1.14 BPM; *R*=0.74; *P*<.001; heart rate pairs: n=12,358) [11]. Overall, the limit of agreement for the Fitbit device was 24 BPM, but its performance was significantly better in patients in sinus rhythm than in those who were not in sinus rhythm (average bias: −0.99 BPM vs −5.02 BPM, respectively; *P*=.02; limits of agreement: 22.9 BPM vs 46.4 BMP, respectively; *P*=.049) [11]. Kroll et al [11,31] also found that the Fitbit was very specific when it detected tachycardia (sensitivity=70%; specificity=99%) [31]. Steinhubl et al [32] demonstrated that manual and automated heart rate readings correlated well (*R*=0.75; measurements: n=111), but limits of agreement were not reported [32]. Weenk et al [4] reported that heart rate readings were generally consistent when compared to the nurse recordings; the limits of agreement for the ViSi Mobile and the HealthPatch were −11.1 to 10.7 BPM and −12.6 to 9.5 BPM, respectively (86 measurements).

#### Sleep

A total of 6 studies used wearables to assess sleep, of which 2 assessed whether wearable readings were reliable. Gallo and Lee [13] found that self-reported sleep correlated with the actigraphy-recorded number of awakenings (*R*=0.53; *P*=.01) [13]. Kroll et al [11,31] found that there was a moderate correlation between wearable-derived sleep duration and questionnaire-derived sleep quality (*R*=0.33; *P*=.03) [31].

#### Respiratory Rate

Of the 8 articles that used different wearables to measure the respiratory rate of patients, 3 assessed the wearables’ accuracy. Breteler et al [30] found that for respiratory rate, the bias was −2.3 breaths per minute, and wide limits of agreement were reported (−15.8 to 11.2 breaths per minute; measurement pairs: n=56,674) [30]. Steinhubl et al [32] reported that there was a strong correlation between wearable and manual respiratory rate readings (*R*=0.83; *P*<.001; measurements: n=111), but limits of agreement were not reported [32]. Weenk et al [4] described wide limits of agreement for respiratory rate based on 86 measurements (ViSi Mobile limits of agreement: −5.5 to 7.9 breaths per minute; HealthPatch limits of agreement: −10.3 to 9.0 breaths per minute) [4].

#### Other Measures

Only 1 study, which was conducted by Weenk et al [4], assessed the accuracy of oxygen saturation and blood pressure readings from ViSi Mobile by comparing them to HealthPatch readings as well as intermittent nurse measurements. From 86 measurements, they found that the automated readings for the systolic blood pressure, diastolic blood pressure, and oxygen saturation had wide limits of agreement (systolic blood pressure: −23.1 to 24.0 mm Hg; diastolic blood pressure: −27.5 to 11.5 mm Hg; oxygen saturation: −3.1% to 3.3%) [4]. Of the 6 articles that used wearables that measured skin temperature, only Steinhubl et al [32] validated the results against a reference standard to conclude that the automated readings were reliable (*R*=0.99; n=112), but bias and limits of agreement were not reported [32]. Of the 3 articles in this review that detected falls by using wearables, only Bloch and colleagues [29] assessed accuracy and found that the Vigi’Fall system had a low sensitivity (37.5%) to fall risk [29].

## Discussion

### Principal Findings

We conducted a systematic review that evaluated the utility of wearable technology in continuously monitoring hospitalized patients for a wide variety of health parameters. Our review focused on the breadth of devices used and the signals measured in hospitalized patients and included consumer, research, and medical-grade devices. There was evidence to support the use of Fitbit, ViSi Mobile, and the HealthPatch to measure heart rate [4,11,31], since the readings were validated against both intermittent and continuous reference standards. This review demonstrated that the validity of the data did not necessarily correlate with the classification of the device because even some medical-grade devices did not perform well and yielded data with wide limits of agreement. We found that only 6 studies validated the accuracy of wearable-derived health data from hospitalized patients by comparing the readings against a reference standard. Overall, the quality of most of these studies was excellent in terms of the reporting of missing data (6/6, 100%) and the use of acceptable accuracy evaluations (5/6, 83%). However, there was a considerable risk of bias in these studies due to the low number of participants in most of the studies (4/6, 67%). Many studies reported wide limits of agreement for other digital biomarkers, such as respiratory rate and blood pressure. Of note, we also found that the majority of studies (8/14, 57%) did not validate the studied device or parameter measured.

Of the various health parameters, the best evidence of validity was in the monitoring of heart rate in hospitalized patients. We also found that, in hospital settings, limits of agreement for medical-grade devices ranged from 16.4 to 21.8 BPM, whereas the limit for a Fitbit consumer device that uses photoplethysmography signals was 24 BPM. Further, during Fitbit-based continuous electrocardiogram monitoring, 73% of the readings were within 5 BPM of electrocardiogram readings. In a systematic review of 158 studies that measured heart rate by using consumer wearable devices, 71% and 51% of Apple Watch (Apple Inc) readings (used in 49 studies) and Fitbit readings (used in 71 studies), respectively, were within 3% of electrocardiogram readings in controlled settings [39]. Moreover, in 3 free-living studies, the wrist-worn Fitbit Charge had a mean absolute error percentage of 10% [39]. A systematic review of wrist-worn devices that measure heart rate via plethysmography found limits of agreement of 8.4 BPM at rest, 30.1 BPM while on a treadmill, and 41.5 BPM while cycling [40]. Overall, our findings found large limits of agreement for all devices, and inpatient results were consistent with the wide limits of agreement found in free-living environments or with activity.

We found that sleep only had a moderate correlation with sleep survey results from inpatient settings the use research and consumer devices. A recent systematic review of Fitbit-based sleep assessments found that readings from more recently developed devices correlated well with polysomnography readings for assessing sleep episodes [41]. It is unclear whether the lower correlation that we found was due to inpatient settings with high nighttime interruptions, patient factors that were perhaps associated with acute illness, or issues with sleep surveys (or a combination of these three factors) [3]. With respect to respiratory rate, 2 studies of 2 medical-grade devices provided limits of agreement. Wider limits of agreement were found in the study that had over 50,000 measurement pairs and used a gold standard (27 breaths per minute) compared to those in the study that had less than 100 measurement pairs and used clinician-reported vitals (13.4-19.3 breaths per minute) [30,32]. Additionally, previous studies found that medical-grade devices were only accurate under laboratory conditions or at-home conditions [42,43]. There was a limited number of studies on oxygen saturation, temperature, blood pressure, and fall risk.

### Limitations and Future Research

There are a few limitations that should be noted for our systematic review. There is a considerable risk of bias, as the number of participants in the studies was low. Further, the studies included were observational in design and had a high degree of heterogeneity in terms of the objectives, populations, and outcomes reported. Thus, the data analysis methods were limited to broad categorization and the extraction of the common themes and trends that emerged from the results. Reports of wearable monitoring from individual studies should be viewed based on their methodological limitations. Although patient adherence has been found to correlate well with patients’ acceptability of wearables devices in inpatient settings, we realize that studying factors such as data loss, the duration of data gaps, and qualitative feedback from nurses and patients would further strengthen the generalizability of the results. Finally, it is important to note that wearable studies are being increasingly performed, and more relevant articles will become increasingly available.

This review also identifies gaps in knowledge that still exist within literature and provides information about what is required for further research. Specifically, the further validation of digital biomarkers by using gold standard comparators, such as polysomnography for assessing sleep and continuous electrocardiogram monitoring for assessing heart rate, is required. Ideally, large participant sample sizes and large numbers of measurement pairs within a population of interest should be used to assess parameters such as vital signs. The use of 2 reference standards to validate each health parameter, such as a heart rate, has also been recommended [44]. Moreover, data that are derived under real life conditions are still needed to better understand the factors that may contribute to between-patient heterogeneity when comparing the accuracy of wearable readings, such as those for patient activity, posture, gait type and velocity, locations of wearables, and patients’ diagnoses (eg, seizures). Future studies can aim to further qualify the process of retrieving data by using wearables to explore other barriers and avenues that might hinder the collection of reliable health information (ie, a weak Bluetooth connectivity, a lack of patient digital health literacy, the added burden that the process of taking wearable readings has on clinicians, the learning curve required to operate a wearable, etc) Finally, while we found that some digital biomarkers appeared to be valid for the monitoring of inpatients via wearables, we were unable to find any studies that supported the use of wearables in inpatient settings to improve clinical outcomes.

### Conclusions

Overall, the assessment of studies in this review suggested that wearable devices show promise for monitoring the heart rate and sleep of patients in hospitals. The results show that many devices were not validated in inpatient settings, and the readings from most of the devices that were validated in such settings had wide limits of agreement. Further research is needed to determine the accuracy of the digital biomarker readings of hospitalized patients and to eventually determine whether wearable devices improve the health outcomes of hospitalized patients.

## Appendix

Multimedia Appendix 1 Search methods.

Multimedia Appendix 2 Modified Consensus-Based Standards for the Selection of Health Status Measurement Instruments (COSMIN) criteria used for the risk of bias assessment.

## Abbreviations

BPM: beats per minute
COSMIN: Consensus-Based Standards for the Selection of Health Status Measurement Instruments
