# Supplementary material for: Evaluating the Validity and Utility of Wearable Technology for Continuously Monitoring Patients in a Hospital Setting: Systematic Review
Source: JMIR Mhealth Uhealth. 2021 Aug 18;9(8):e17411. doi: 10.2196/17411 (PMC8411322; doi:10.2196/17411)
Supplement: Multimedia Appendix 1 [file mhealth_v9i8e17411_app1.docx]

## Search Methods for: Vikas Patel, Robert Wu, Ani Orchanian-Cheff. Evaluating the validity and utility of wearable technology to continuously monitor patients in a hospital setting: a systematic review

September 5, 2018

## Ani Orchanian-Cheff, MISt ([ani.orchanian-cheff@uhn.ca](mailto:ani.orchanian-cheff@uhn.ca)), Library and Information Services, University Health Network [
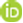
](https://orcid.org/0000-0002-9943-2692) <https://orcid.org/0000-0002-9943-2692>

**Search Methods:**

A comprehensive search strategy was developed by a medical librarian (AOC) to identify articles on the three main concepts of our question: wearables + monitoring + inpatients.

The initial search strategy was developed for Ovid Medline using a combination of database-specific subject headings and text words. Additional key words were generated through input from subject specialists on the team and the revised search strategy was customized for each database.

Searches of the following databases were executed on August 16^th^, 2018: Ovid MEDLINE, Ovid MEDLINE Epub Ahead of Print and In-Process & Other Non-Indexed Citations. Cochrane Database of Systematic Reviews, Cochrane Central Register of Controlled Trials (CENTRAL), Health Technology Assessment (HTA) database (Ovid), and CINAHL with Full Text. The search in Ovid Embase was not executed until September 5, 2018 due to issues with the vendor’s August database reload.

There were no restrictions on publication period. Animal-only studies were excluded where applicable. Limits were imposed for English language and adult population. No other limits were applied. See Appendix I for database search strategies.

Additional search methods included reviewing cited references of eligible studies through Web of Science (May 6^th^, 2019), and reference lists of eligible studies.

**Appendix I: Database Search Strategies**

Database: Ovid MEDLINE(R) <1946 to August Week 2 2018>

Search Strategy:

--------------------------------------------------------------------------------

1 Wearable Electronic Devices/ (427)

2 Fitness Trackers/ (173)

3 exp Clothing/ (25375)

4 exp Textiles/ (12261)

5 Jewelry/ (267)

6 or/3-5 (36938)

7 Wireless Technology/ (2824)

8 exp Telemedicine/ (23273)

9 exp TELEMETRY/ (11866)

10 Biosensing Techniques/ (34150)

11 or/7-10 (70442)

12 6 and 11 (346)

13 wearable*.mp. (4376)

14 (smart adj5 fabric?).mp. (23)

15 (smart adj5 cloth*).mp. (32)

16 (smart adj5 textile*).mp. (85)

17 (smart adj5 patch??).mp. (20)

18 (smart adj5 glove?).mp. (7)

19 (smart adj5 shoe?).mp. (11)

20 (smart adj5 accessor*).mp. (4)

21 (smart adj5 jewel*).mp. (0)

22 (smart adj5 wrist*).mp. (5)

23 (smart adj5 watch*).mp. (36)

24 smartwatch*.mp. (54)

25 fitbit?.mp. (151)

26 (smart adj5 neckless*).mp. (0)

27 (smart adj5 belt?).mp. (5)

28 (smart adj5 skin).mp. (28)

29 (smart adj5 armband*).mp. (1)

30 (smart adj5 shirt*).mp. (9)

31 (smart adj5 p?jama*).mp. (0)

32 (smart adj5 glasses).mp. (25)

33 (smart adj5 vest?).mp. (1)

34 (smart adj5 garment*).mp. (31)

35 (smart adj5 fashion*).mp. (10)

36 (smart adj5 footwear).mp. (0)

37 (smart adj5 eyewear).mp. (0)

38 (smart adj5 body).mp. (32)

39 (smart adj5 sensor*).mp. (329)

40 (smart adj5 biosensor*).mp. (51)

41 (intelligent adj5 biosensor*).mp. (12)

42 (wireless adj5 biosensor*).mp. (46)

43 (wireless adj2 body).mp. (276)

44 WBAN?.mp. (131)

45 wireless sensor network*.mp. (522)

46 (body adj2 network*).mp. (525)

47 (body adj2 sensor*).mp. (726)

48 personal area network*.mp. (31)

49 (context-aware adj2 system?).mp. (42)

50 (mhealth adj2 patch??).mp. (0)

51 (m-health adj2 patch??).mp. (0)

52 (textile adj3 transducer*).mp. (1)

53 (sensor? adj5 garment*).mp. (36)

54 (biosensor? adj5 garment*).mp. (0)

55 (sensor? adj5 cloth*).mp. (48)

56 (biosensor? adj5 cloth*).mp. (2)

57 (sensor? adj5 fabric*).mp. (1540)

58 (biosensor? adj5 fabric*).mp. (1104)

59 (bionic adj2 glove*).mp. (2)

60 (high-tech adj3 textile*).mp. (1)

61 (sensor? adj5 textile*).mp. (108)

62 (biosensor? adj5 textile*).mp. (7)

63 (wear* adj3 body).mp. (534)

64 (wear* adj5 device*).mp. (2356)

65 (wear* adj5 instrument*).mp. (162)

66 (wear* adj5 smart).mp. (123)

67 (wear* adj5 track*).mp. (257)

68 (wear* adj5 system*).mp. (1461)

69 (worn adj3 body).mp. (369)

70 (worn adj5 track*).mp. (33)

71 (worn adj5 smart).mp. (5)

72 (worn adj5 sensor*).mp. (327)

73 (worn adj5 biosensor*).mp. (6)

74 (tech* adj2 tog?).mp. (4)

75 (fashion* adj3 electronic*).mp. (16)

76 (fashion* adj3 tech).mp. (1)

77 (fashion* adj3 technolog*).mp. (36)

78 (connected adj3 cloth*).mp. (4)

79 (bluetooth adj3 cloth*).mp. (0)

80 (internet adj3 cloth*).mp. (1)

81 (digital adj2 cloth*).mp. (2)

82 (advanced adj2 textile*).mp. (10)

83 (electronic* adj2 textile*).mp. (71)

84 e-textile*.mp. (37)

85 (advanced adj2 fabric*).mp. (95)

86 (electronic* adj3 cloth*).mp. (23)

87 (WATCH adj2 society).mp. (1)

88 (life adj2 shirt).mp. (0)

89 (life adj2 vest).mp. (18)

90 body-mount*.mp. (74)

91 (electronic* adj2 skin*).mp. (81)

92 (electronic* adj2 patch??).mp. (17)

93 (activit* adj2 track*).mp. (641)

94 (fitness* adj2 track*).mp. (242)

95 (physical* adj2 track*).mp. (192)

96 or/1-2,12-95 (12670)

97 exp Monitoring, Physiologic/ (159501)

98 monitor*.mp. (756223)

99 surveill*.mp. (177911)

100 (continu* adj3 measur*).mp. (15247)

101 (continu* adj3 record*).mp. (9710)

102 (continu* adj3 detect*).mp. (2034)

103 (continu* adj3 sensor?).mp. (881)

104 (continu* adj3 sensing).mp. (143)

105 (continu* adj3 scan*).mp. (764)

106 (continu* adj3 observation?).mp. (1953)

107 Somnograph*.mp. (62)

108 Polysomnograph*.mp. (22779)

109 Actigraph*.mp. (5668)

110 Acceleromet*.mp. (11266)

111 Capnomet*.mp. (504)

112 Electrocardiograph*.mp. (201620)

113 (track* adj3 vital sign*).mp. (9)

114 (measure* adj3 vital sign*).mp. (715)

115 neuromonitor*.mp. (1199)

116 tocograph*.mp. (168)

117 Cardiotocograph*.mp. (2816)

118 or/97-117 (1147265)

119 96 and 118 (5218)

120 Inpatients/ (18345)

121 exp Hospitalization/ (210267)

122 exp Hospital Units/ (98573)

123 exp Hospitals/ (253976)

124 inpatient*.mp. (88760)

125 in-patient*.mp. (1341851)

126 hospital*.mp. (1279250)

127 ward?.mp. (46006)

128 medical service.tw. (6259)

129 (medical adj2 unit?).mp. (3896)

130 surgical service.tw. (969)

131 (surgical adj2 unit?).mp. (3791)

132 (H?emodialysis adj2 Unit*).mp. (2333)

133 (Dialysis adj2 Unit?).mp. (2062)

134 ICU?.mp. (40965)

135 intensive care.mp. (134304)

136 (intensive therapy adj2 unit?).mp. (583)

137 (intensive treatment adj2 unit?).mp. (59)

138 (close attention adj2 unit?).mp. (2)

139 (Burn adj2 Unit?).mp. (3140)

140 CCU?.mp. (2734)

141 (cardiolog* adj2 unit?).mp. (530)

142 (coronary adj2 unit?).mp. (6966)

143 coronary care.mp. (7140)

144 GICU?.mp. (23)

145 (geriatric adj2 unit?).mp. (1194)

146 (gerontolog* adj2 unit?).mp. (18)

147 Recovery Room?.mp. (3579)

148 (respiratory care adj2 unit?).mp. (665)

149 (special care adj2 unit?).mp. (1072)

150 (nurs* adj2 station?).mp. (457)

151 (nurs* adj2 unit?).mp. (3399)

152 operating room?.mp. (33888)

153 (self-care adj2 unit?).mp. (145)

154 (Minimal Care adj2 unit?).mp. (18)

155 (high dependency adj2 unit?).mp. (517)

156 (palliative care adj2 unit?).mp. (1310)

157 (psychiatr* adj2 unit?).mp. (3339)

158 (stroke adj2 unit?).mp. (2643)

159 or/120-158 (2642813)

160 119 and 159 (559)

161 limit 160 to "all child (0 to 18 years)" (48)

162 limit 160 to "all adult (19 plus years)" (251)

163 160 not 161 (511)

164 162 or 163 (541)

165 limit 164 to english language (531)

166 animals/ not (animals/ and humans/) (4453466)

167 165 not 166 (528)

Database: Ovid MEDLINE(R) Epub Ahead of Print and In-Process & Other Non-Indexed Citations <August 15, 2018>

Search Strategy:

--------------------------------------------------------------------------------

1 wearable*.mp. (2974)

2 (smart adj5 fabric?).mp. (23)

3 (smart adj5 cloth*).mp. (27)

4 (smart adj5 textile*).mp. (79)

5 (smart adj5 patch??).mp. (16)

6 (smart adj5 glove?).mp. (8)

7 (smart adj5 shoe?).mp. (2)

8 (smart adj5 accessor*).mp. (0)

9 (smart adj5 jewel*).mp. (1)

10 (smart adj5 wrist*).mp. (13)

11 (smart adj5 watch*).mp. (41)

12 smartwatch*.mp. (58)

13 fitbit?.mp. (175)

14 (smart adj5 neckless*).mp. (0)

15 (smart adj5 belt?).mp. (1)

16 (smart adj5 skin).mp. (27)

17 (smart adj5 armband*).mp. (1)

18 (smart adj5 shirt*).mp. (6)

19 (smart adj5 p?jama*).mp. (1)

20 (smart adj5 glasses).mp. (20)

21 (smart adj5 vest?).mp. (1)

22 (smart adj5 garment*).mp. (14)

23 (smart adj5 fashion*).mp. (2)

24 (smart adj5 footwear).mp. (1)

25 (smart adj5 eyewear).mp. (0)

26 (smart adj5 body).mp. (18)

27 (smart adj5 sensor*).mp. (370)

28 (smart adj5 biosensor*).mp. (18)

29 (intelligent adj5 biosensor*).mp. (3)

30 (wireless adj5 biosensor*).mp. (13)

31 (wireless adj2 body).mp. (97)

32 WBAN?.mp. (52)

33 wireless sensor network*.mp. (975)

34 (body adj2 network*).mp. (167)

35 (body adj2 sensor*).mp. (199)

36 personal area network*.mp. (17)

37 (context-aware adj2 system?).mp. (15)

38 (mhealth adj2 patch??).mp. (0)

39 (m-health adj2 patch??).mp. (0)

40 (textile adj3 transducer*).mp. (1)

41 (sensor? adj5 garment*).mp. (12)

42 (biosensor? adj5 garment*).mp. (0)

43 (sensor? adj5 cloth*).mp. (8)

44 (biosensor? adj5 cloth*).mp. (3)

45 (sensor? adj5 fabric*).mp. (1438)

46 (biosensor? adj5 fabric*).mp. (201)

47 (bionic adj2 glove*).mp. (0)

48 (high-tech adj3 textile*).mp. (1)

49 (sensor? adj5 textile*).mp. (51)

50 (biosensor? adj5 textile*).mp. (7)

51 (wear* adj3 body).mp. (90)

52 (wear* adj5 device*).mp. (1213)

53 (wear* adj5 instrument*).mp. (18)

54 (wear* adj5 smart).mp. (137)

55 (wear* adj5 track*).mp. (159)

56 (wear* adj5 system*).mp. (450)

57 (worn adj3 body).mp. (77)

58 (worn adj5 track*).mp. (24)

59 (worn adj5 smart).mp. (5)

60 (worn adj5 sensor*).mp. (98)

61 (worn adj5 biosensor*).mp. (0)

62 (tech* adj2 tog?).mp. (0)

63 (fashion* adj3 electronic*).mp. (6)

64 (fashion* adj3 tech).mp. (0)

65 (fashion* adj3 technolog*).mp. (7)

66 (connected adj3 cloth*).mp. (2)

67 (bluetooth adj3 cloth*).mp. (0)

68 (internet adj3 cloth*).mp. (0)

69 (digital adj2 cloth*).mp. (0)

70 (advanced adj2 textile*).mp. (7)

71 (electronic* adj2 textile*).mp. (81)

72 e-textile*.mp. (30)

73 (advanced adj2 fabric*).mp. (128)

74 (electronic* adj3 cloth*).mp. (10)

75 (WATCH adj2 society).mp. (0)

76 (life adj2 shirt).mp. (0)

77 (life adj2 vest).mp. (5)

78 body-mount*.mp. (15)

79 (electronic* adj2 skin*).mp. (187)

80 (electronic* adj2 patch??).mp. (4)

81 (activit* adj2 track*).mp. (304)

82 (fitness* adj2 track*).mp. (79)

83 (physical* adj2 track*).mp. (70)

84 or/1-83 (7204)

85 monitor*.mp. (94370)

86 surveill*.mp. (20138)

87 (continu* adj3 measur*).mp. (2173)

88 (continu* adj3 record*).mp. (893)

89 (continu* adj3 detect*).mp. (407)

90 (continu* adj3 sensor?).mp. (222)

91 (continu* adj3 sensing).mp. (57)

92 (continu* adj3 scan*).mp. (247)

93 (continu* adj3 observation?).mp. (325)

94 Somnograph*.mp. (3)

95 Polysomnograph*.mp. (1772)

96 Actigraph*.mp. (864)

97 Acceleromet*.mp. (2648)

98 Capnomet*.mp. (36)

99 Electrocardiograph*.mp. (3066)

100 (track* adj3 vital sign*).mp. (2)

101 (measure* adj3 vital sign*).mp. (123)

102 neuromonitor*.mp. (323)

103 tocograph*.mp. (6)

104 Cardiotocograph*.mp. (143)

105 or/85-104 (121837)

106 84 and 105 (2096)

107 inpatient*.mp. (11577)

108 in-patient*.mp. (166136)

109 hospital*.mp. (142294)

110 ward?.mp. (6840)

111 medical service.tw. (899)

112 (medical adj2 unit?).mp. (471)

113 surgical service.tw. (75)

114 (surgical adj2 unit?).mp. (414)

115 (H?emodialysis adj2 Unit*).mp. (117)

116 (Dialysis adj2 Unit?).mp. (159)

117 ICU?.mp. (7972)

118 intensive care.mp. (16487)

119 (intensive therapy adj2 unit?).mp. (39)

120 (intensive treatment adj2 unit?).mp. (3)

121 (close attention adj2 unit?).mp. (0)

122 (Burn adj2 Unit?).mp. (178)

123 CCU?.mp. (304)

124 (cardiolog* adj2 unit?).mp. (77)

125 (coronary adj2 unit?).mp. (258)

126 coronary care.mp. (270)

127 GICU?.mp. (5)

128 (geriatric adj2 unit?).mp. (91)

129 (gerontolog* adj2 unit?).mp. (2)

130 Recovery Room?.mp. (326)

131 (respiratory care adj2 unit?).mp. (12)

132 (special care adj2 unit?).mp. (83)

133 (nurs* adj2 station?).mp. (51)

134 (nurs* adj2 unit?).mp. (322)

135 operating room?.mp. (3088)

136 (self-care adj2 unit?).mp. (1)

137 (Minimal Care adj2 unit?).mp. (1)

138 (high dependency adj2 unit?).mp. (87)

139 (palliative care adj2 unit?).mp. (191)

140 (psychiatr* adj2 unit?).mp. (421)

141 (stroke adj2 unit?).mp. (340)

142 or/107-141 (302748)

143 106 and 142 (170)

Database: Embase <1974 to 2018 September 4>

Search Strategy:

--------------------------------------------------------------------------------

1 exp biosensor/ (34926)

2 activity tracker/ (314)

3 wireless communication/ (4223)

4 exp telemedicine/ (31969)

5 exp telemetry/ (23296)

6 exp electronic device/ (259072)

7 or/1-6 (343211)

8 exp clothing/ (36236)

9 textile/ (4497)

10 or/8-9 (40005)

11 7 and 10 (1074)

12 wearable*.mp. (7682)

13 (smart adj5 fabric?).mp. (33)

14 (smart adj5 cloth*).mp. (47)

15 (smart adj5 textile*).mp. (119)

16 (smart adj5 patch??).mp. (50)

17 (smart adj5 glove?).mp. (17)

18 (smart adj5 shoe?).mp. (22)

19 (smart adj5 accessor*).mp. (6)

20 (smart adj5 jewel*).mp. (0)

21 (smart adj5 wrist*).mp. (17)

22 (smart adj5 watch*).mp. (107)

23 smartwatch*.mp. (135)

24 fitbit?.mp. (476)

25 (smart adj5 neckless*).mp. (0)

26 (smart adj5 belt?).mp. (8)

27 (smart adj5 skin).mp. (52)

28 (smart adj5 armband*).mp. (2)

29 (smart adj5 shirt*).mp. (27)

30 (smart adj5 p?jama*).mp. (0)

31 (smart adj5 glasses).mp. (53)

32 (smart adj5 vest?).mp. (2)

33 (smart adj5 garment*).mp. (49)

34 (smart adj5 fashion*).mp. (15)

35 (smart adj5 footwear).mp. (2)

36 (smart adj5 eyewear).mp. (1)

37 (smart adj5 body).mp. (66)

38 (smart adj5 sensor*).mp. (573)

39 (smart adj5 biosensor*).mp. (68)

40 (intelligent adj5 biosensor*).mp. (17)

41 (wireless adj5 biosensor*).mp. (66)

42 (wireless adj2 body).mp. (429)

43 WBAN?.mp. (204)

44 wireless sensor network*.mp. (899)

45 (body adj2 network*).mp. (803)

46 (body adj2 sensor*).mp. (1156)

47 personal area network*.mp. (49)

48 (context-aware adj2 system?).mp. (67)

49 (mhealth adj2 patch??).mp. (0)

50 (m-health adj2 patch??).mp. (0)

51 (textile adj3 transducer*).mp. (2)

52 (sensor? adj5 garment*).mp. (62)

53 (biosensor? adj5 garment*).mp. (0)

54 (sensor? adj5 cloth*).mp. (81)

55 (biosensor? adj5 cloth*).mp. (5)

56 (sensor? adj5 fabric*).mp. (2212)

57 (biosensor? adj5 fabric*).mp. (1388)

58 (bionic adj2 glove*).mp. (11)

59 (high-tech adj3 textile*).mp. (1)

60 (sensor? adj5 textile*).mp. (174)

61 (biosensor? adj5 textile*).mp. (18)

62 (wear* adj3 body).mp. (687)

63 (wear* adj5 device*).mp. (3544)

64 (wear* adj5 instrument*).mp. (215)

65 (wear* adj5 smart).mp. (218)

66 (wear* adj5 track*).mp. (447)

67 (wear* adj5 system*).mp. (2217)

68 (worn adj3 body).mp. (592)

69 (worn adj5 track*).mp. (67)

70 (worn adj5 smart).mp. (16)

71 (worn adj5 sensor*).mp. (623)

72 (worn adj5 biosensor*).mp. (9)

73 (tech* adj2 tog?).mp. (3)

74 (fashion* adj3 electronic*).mp. (38)

75 (fashion* adj3 tech).mp. (1)

76 (fashion* adj3 technolog*).mp. (73)

77 (connected adj3 cloth*).mp. (10)

78 (bluetooth adj3 cloth*).mp. (0)

79 (internet adj3 cloth*).mp. (2)

80 (digital adj2 cloth*).mp. (2)

81 (advanced adj2 textile*).mp. (15)

82 (electronic* adj2 textile*).mp. (104)

83 e-textile*.mp. (50)

84 (advanced adj2 fabric*).mp. (148)

85 (electronic* adj3 cloth*).mp. (33)

86 (WATCH adj2 society).mp. (1)

87 (life adj2 shirt).mp. (3)

88 (life adj2 vest).mp. (60)

89 body-mount*.mp. (94)

90 (electronic* adj2 skin*).mp. (133)

91 (electronic* adj2 patch??).mp. (27)

92 (activit* adj2 track*).mp. (1314)

93 (fitness* adj2 track*).mp. (182)

94 (physical* adj2 track*).mp. (281)

95 or/11-94 (20872)

96 exp patient monitoring/ (185652)

97 monitoring/ (154326)

98 biological monitoring/ (15944)

99 drug monitoring/ (49478)

100 exp hemodynamic monitoring/ (51289)

101 exp physiologic monitoring/ (4968)

102 monitor*.mp. (1184340)

103 surveill*.mp. (248272)

104 (continu* adj3 measur*).mp. (22625)

105 (continu* adj3 record*).mp. (14004)

106 (continu* adj3 detect*).mp. (3019)

107 (continu* adj3 sensor?).mp. (1394)

108 (continu* adj3 sensing).mp. (228)

109 (continu* adj3 scan*).mp. (1242)

110 (continu* adj3 observation?).mp. (2779)

111 Somnograph*.mp. (125)

112 Polysomnograph*.mp. (37926)

113 Actigraph*.mp. (9089)

114 Acceleromet*.mp. (18537)

115 Capnomet*.mp. (2311)

116 Electrocardiograph*.mp. (149976)

117 (track* adj3 vital sign*).mp. (21)

118 (measure* adj3 vital sign*).mp. (1254)

119 neuromonitor*.mp. (4818)

120 tocograph*.mp. (154)

121 Cardiotocograph*.mp. (5168)

122 or/96-121 (1603349)

123 95 and 122 (8246)

124 exp hospital patient/ (144455)

125 exp hospital/ (993139)

126 exp "hospital subdivisions and components"/ (443146)

127 hospitalization/ (307927)

128 inpatient*.mp. (148036)

129 in-patient*.mp. (2258480)

130 hospital*.mp. (2194887)

131 ward?.mp. (197698)

132 medical service.tw. (7977)

133 (medical adj2 unit?).mp. (6789)

134 surgical service.tw. (1280)

135 (surgical adj2 unit?).mp. (5832)

136 (H?emodialysis adj2 Unit*).mp. (1771)

137 (Dialysis adj2 Unit?).mp. (3109)

138 ICU?.mp. (98875)

139 intensive care.mp. (302925)

140 (intensive therapy adj2 unit?).mp. (765)

141 (intensive treatment adj2 unit?).mp. (109)

142 (close attention adj2 unit?).mp. (2)

143 (Burn adj2 Unit?).mp. (2568)

144 CCU?.mp. (4309)

145 (cardiolog* adj2 unit?).mp. (1203)

146 (coronary adj2 unit?).mp. (11929)

147 coronary care.mp. (12200)

148 GICU?.mp. (55)

149 (geriatric adj2 unit?).mp. (1962)

150 (gerontolog* adj2 unit?).mp. (26)

151 Recovery Room?.mp. (7585)

152 (respiratory care adj2 unit?).mp. (217)

153 (special care adj2 unit?).mp. (1443)

154 (nurs* adj2 station?).mp. (649)

155 (nurs* adj2 unit?).mp. (4527)

156 operating room?.mp. (52708)

157 (self-care adj2 unit?).mp. (33)

158 (Minimal Care adj2 unit?).mp. (14)

159 (high dependency adj2 unit?).mp. (1231)

160 (palliative care adj2 unit?).mp. (2582)

161 (psychiatr* adj2 unit?).mp. (5330)

162 (stroke adj2 unit?).mp. (7332)

163 or/124-162 (4398856)

164 123 and 163 (1346)

165 limit 164 to (embryo <first trimester> or infant <to one year> or child <unspecified age> or preschool child <1 to 6 years> or school child <7 to 12 years> or adolescent <13 to 17 years>) (73)

166 limit 164 to (adult <18 to 64 years> or aged <65+ years>) (544)

167 164 not 165 (1273)

168 166 or 167 (1305)

169 (exp animals/ or exp animal experimentation/ or nonhuman/) not ((exp animals/ or exp animal experimentation/ or nonhuman/) and exp human/) (5977327)

170 168 not 169 (1292)

171 limit 170 to english language (1271)

172 remove duplicates from 171 (1226)

Database: Cochrane Central Register of Controlled Trials

Search Strategy:

--------------------------------------------------------------------------------

1 Wearable Electronic Devices/ (7)

2 Fitness Trackers/ (31)

3 exp Clothing/ (1149)

4 exp Textiles/ (206)

5 Jewelry/ (3)

6 or/3-5 (1318)

7 Wireless Technology/ (33)

8 exp Telemedicine/ (1835)

9 exp TELEMETRY/ (254)

10 Biosensing Techniques/ (48)

11 or/7-10 (2103)

12 6 and 11 (4)

13 wearable*.mp. (469)

14 (smart adj5 fabric?).mp. (0)

15 (smart adj5 cloth*).mp. (0)

16 (smart adj5 textile*).mp. (1)

17 (smart adj5 patch??).mp. (0)

18 (smart adj5 glove?).mp. (9)

19 (smart adj5 shoe?).mp. (1)

20 (smart adj5 accessor*).mp. (1)

21 (smart adj5 jewel*).mp. (0)

22 (smart adj5 wrist*).mp. (3)

23 (smart adj5 watch*).mp. (16)

24 smartwatch*.mp. (12)

25 fitbit?.mp. (175)

26 (smart adj5 neckless*).mp. (0)

27 (smart adj5 belt?).mp. (0)

28 (smart adj5 skin).mp. (10)

29 (smart adj5 armband*).mp. (0)

30 (smart adj5 shirt*).mp. (0)

31 (smart adj5 p?jama*).mp. (0)

32 (smart adj5 glasses).mp. (1)

33 (smart adj5 vest?).mp. (0)

34 (smart adj5 garment*).mp. (1)

35 (smart adj5 fashion*).mp. (1)

36 (smart adj5 footwear).mp. (1)

37 (smart adj5 eyewear).mp. (0)

38 (smart adj5 body).mp. (8)

39 (smart adj5 sensor*).mp. (15)

40 (smart adj5 biosensor*).mp. (0)

41 (intelligent adj5 biosensor*).mp. (0)

42 (wireless adj5 biosensor*).mp. (0)

43 (wireless adj2 body).mp. (3)

44 WBAN?.mp. (0)

45 wireless sensor network*.mp. (0)

46 (body adj2 network*).mp. (18)

47 (body adj2 sensor*).mp. (54)

48 personal area network*.mp. (0)

49 (context-aware adj2 system?).mp. (1)

50 (mhealth adj2 patch??).mp. (0)

51 (m-health adj2 patch??).mp. (0)

52 (textile adj3 transducer*).mp. (0)

53 (sensor? adj5 garment*).mp. (4)

54 (biosensor? adj5 garment*).mp. (0)

55 (sensor? adj5 cloth*).mp. (2)

56 (biosensor? adj5 cloth*).mp. (0)

57 (sensor? adj5 fabric*).mp. (4)

58 (biosensor? adj5 fabric*).mp. (1)

59 (bionic adj2 glove*).mp. (0)

60 (high-tech adj3 textile*).mp. (0)

61 (sensor? adj5 textile*).mp. (0)

62 (biosensor? adj5 textile*).mp. (0)

63 (wear* adj3 body).mp. (47)

64 (wear* adj5 device*).mp. (401)

65 (wear* adj5 instrument*).mp. (19)

66 (wear* adj5 smart).mp. (11)

67 (wear* adj5 track*).mp. (98)

68 (wear* adj5 system*).mp. (179)

69 (worn adj3 body).mp. (54)

70 (worn adj5 track*).mp. (19)

71 (worn adj5 smart).mp. (1)

72 (worn adj5 sensor*).mp. (48)

73 (worn adj5 biosensor*).mp. (0)

74 (tech* adj2 tog?).mp. (1)

75 (fashion* adj3 electronic*).mp. (7)

76 (fashion* adj3 tech).mp. (0)

77 (fashion* adj3 technolog*).mp. (3)

78 (connected adj3 cloth*).mp. (0)

79 (bluetooth adj3 cloth*).mp. (0)

80 (internet adj3 cloth*).mp. (0)

81 (digital adj2 cloth*).mp. (1)

82 (advanced adj2 textile*).mp. (0)

83 (electronic* adj2 textile*).mp. (0)

84 e-textile*.mp. (0)

85 (advanced adj2 fabric*).mp. (0)

86 (electronic* adj3 cloth*).mp. (2)

87 (WATCH adj2 society).mp. (0)

88 (life adj2 shirt).mp. (0)

89 (life adj2 vest).mp. (0)

90 body-mount*.mp. (4)

91 (electronic* adj2 skin*).mp. (11)

92 (electronic* adj2 patch??).mp. (4)

93 (activit* adj2 track*).mp. (244)

94 (fitness* adj2 track*).mp. (48)

95 (physical* adj2 track*).mp. (96)

96 or/1-2,12-95 (1477)

97 exp Monitoring, Physiologic/ (11176)

98 monitor*.mp. (67748)

99 surveill*.mp. (6183)

100 (continu* adj3 measur*).mp. (2451)

101 (continu* adj3 record*).mp. (1494)

102 (continu* adj3 detect*).mp. (190)

103 (continu* adj3 sensor?).mp. (122)

104 (continu* adj3 sensing).mp. (7)

105 (continu* adj3 scan*).mp. (69)

106 (continu* adj3 observation?).mp. (274)

107 Somnograph*.mp. (18)

108 Polysomnograph*.mp. (3603)

109 Actigraph*.mp. (1601)

110 Acceleromet*.mp. (2383)

111 Capnomet*.mp. (200)

112 Electrocardiograph*.mp. (12116)

113 (track* adj3 vital sign*).mp. (4)

114 (measure* adj3 vital sign*).mp. (800)

115 neuromonitor*.mp. (132)

116 tocograph*.mp. (11)

117 Cardiotocograph*.mp. (332)

118 or/97-117 (91614)

119 96 and 118 (608)

120 Inpatients/ (811)

121 exp Hospitalization/ (12123)

122 exp Hospital Units/ (3498)

123 exp Hospitals/ (3249)

124 inpatient*.mp. (11381)

125 in-patient*.mp. (717141)

126 hospital*.mp. (122657)

127 ward?.mp. (8671)

128 medical service.tw. (282)

129 (medical adj2 unit?).mp. (553)

130 surgical service.tw. (37)

131 (surgical adj2 unit?).mp. (415)

132 (H?emodialysis adj2 Unit*).mp. (158)

133 (Dialysis adj2 Unit?).mp. (188)

134 ICU?.mp. (7956)

135 intensive care.mp. (16554)

136 (intensive therapy adj2 unit?).mp. (33)

137 (intensive treatment adj2 unit?).mp. (4)

138 (close attention adj2 unit?).mp. (0)

139 (Burn adj2 Unit?).mp. (119)

140 CCU?.mp. (183)

141 (cardiolog* adj2 unit?).mp. (62)

142 (coronary adj2 unit?).mp. (570)

143 coronary care.mp. (566)

144 GICU?.mp. (0)

145 (geriatric adj2 unit?).mp. (159)

146 (gerontolog* adj2 unit?).mp. (1)

147 Recovery Room?.mp. (2217)

148 (respiratory care adj2 unit?).mp. (22)

149 (special care adj2 unit?).mp. (108)

150 (nurs* adj2 station?).mp. (26)

151 (nurs* adj2 unit?).mp. (375)

152 operating room?.mp. (3160)

153 (self-care adj2 unit?).mp. (12)

154 (Minimal Care adj2 unit?).mp. (4)

155 (high dependency adj2 unit?).mp. (52)

156 (palliative care adj2 unit?).mp. (90)

157 (psychiatr* adj2 unit?).mp. (213)

158 (stroke adj2 unit?).mp. (639)

159 or/120-158 (741826)

160 119 and 159 (356)

161 limit 160 to english language (208)

162 remove duplicates from 161 (206)

Database: Cochrane Database of Systematic Reviews <2005 to Present>

Search Strategy:

--------------------------------------------------------------------------------

1 wearable*.tw. (15)

2 (smart adj5 fabric?).tw. (0)

3 (smart adj5 cloth*).tw. (0)

4 (smart adj5 textile*).tw. (0)

5 (smart adj5 patch??).tw. (0)

6 (smart adj5 glove?).tw. (0)

7 (smart adj5 shoe?).tw. (0)

8 (smart adj5 accessor*).tw. (0)

9 (smart adj5 jewel*).tw. (0)

10 (smart adj5 wrist*).tw. (0)

11 (smart adj5 watch*).tw. (0)

12 smartwatch*.tw. (0)

13 fitbit?.tw. (2)

14 (smart adj5 neckless*).tw. (0)

15 (smart adj5 belt?).tw. (0)

16 (smart adj5 skin).tw. (1)

17 (smart adj5 armband*).tw. (0)

18 (smart adj5 shirt*).tw. (0)

19 (smart adj5 p?jama*).tw. (0)

20 (smart adj5 glasses).tw. (0)

21 (smart adj5 vest?).tw. (1)

22 (smart adj5 garment*).tw. (0)

23 (smart adj5 fashion*).tw. (0)

24 (smart adj5 footwear).tw. (0)

25 (smart adj5 eyewear).tw. (0)

26 (smart adj5 body).tw. (0)

27 (smart adj5 sensor*).tw. (1)

28 (smart adj5 biosensor*).tw. (0)

29 (intelligent adj5 biosensor*).tw. (0)

30 (wireless adj5 biosensor*).tw. (0)

31 (wireless adj2 body).tw. (0)

32 WBAN?.tw. (0)

33 wireless sensor network*.tw. (0)

34 (body adj2 network*).tw. (0)

35 (body adj2 sensor*).tw. (8)

36 personal area network*.tw. (0)

37 (context-aware adj2 system?).tw. (0)

38 (mhealth adj2 patch??).tw. (0)

39 (m-health adj2 patch??).tw. (0)

40 (textile adj3 transducer*).tw. (0)

41 (sensor? adj5 garment*).tw. (1)

42 (biosensor? adj5 garment*).tw. (0)

43 (sensor? adj5 cloth*).tw. (0)

44 (biosensor? adj5 cloth*).tw. (0)

45 (sensor? adj5 fabric*).tw. (1)

46 (biosensor? adj5 fabric*).tw. (0)

47 (bionic adj2 glove*).tw. (0)

48 (high-tech adj3 textile*).tw. (0)

49 (sensor? adj5 textile*).tw. (0)

50 (biosensor? adj5 textile*).tw. (0)

51 (wear* adj3 body).tw. (3)

52 (wear* adj5 device*).tw. (29)

53 (wear* adj5 instrument*).tw. (2)

54 (wear* adj5 smart).tw. (2)

55 (wear* adj5 track*).tw. (2)

56 (wear* adj5 system*).tw. (8)

57 (worn adj3 body).tw. (14)

58 (worn adj5 track*).tw. (2)

59 (worn adj5 smart).tw. (0)

60 (worn adj5 sensor*).tw. (7)

61 (worn adj5 biosensor*).tw. (0)

62 (tech* adj2 tog?).tw. (0)

63 (fashion* adj3 electronic*).tw. (0)

64 (fashion* adj3 tech).tw. (0)

65 (fashion* adj3 technolog*).tw. (0)

66 (connected adj3 cloth*).tw. (0)

67 (bluetooth adj3 cloth*).tw. (0)

68 (internet adj3 cloth*).tw. (0)

69 (digital adj2 cloth*).tw. (0)

70 (advanced adj2 textile*).tw. (0)

71 (electronic* adj2 textile*).tw. (0)

72 e-textile*.tw. (0)

73 (advanced adj2 fabric*).tw. (0)

74 (electronic* adj3 cloth*).tw. (0)

75 (WATCH adj2 society).tw. (0)

76 (life adj2 shirt).tw. (0)

77 (life adj2 vest).tw. (0)

78 body-mount*.tw. (2)

79 (electronic* adj2 skin*).tw. (0)

80 (electronic* adj2 patch??).tw. (0)

81 (activit* adj2 track*).tw. (4)

82 (fitness* adj2 track*).tw. (1)

83 (physical* adj2 track*).tw. (0)

84 or/1-83 (75)

85 monitor*.tw. (3064)

86 surveill*.tw. (734)

87 (continu* adj3 measur*).tw. (2150)

88 (continu* adj3 record*).tw. (231)

89 (continu* adj3 detect*).tw. (38)

90 (continu* adj3 sensor?).tw. (9)

91 (continu* adj3 sensing).tw. (0)

92 (continu* adj3 scan*).tw. (6)

93 (continu* adj3 observation?).tw. (29)

94 Somnograph*.tw. (0)

95 Polysomnograph*.tw. (68)

96 Actigraph*.tw. (38)

97 Acceleromet*.tw. (63)

98 Capnomet*.tw. (3)

99 Electrocardiograph*.tw. (115)

100 (track* adj3 vital sign*).tw. (0)

101 (measure* adj3 vital sign*).tw. (14)

102 neuromonitor*.tw. (4)

103 tocograph*.tw. (2)

104 Cardiotocograph*.tw. (55)

105 or/85-104 (4947)

106 84 and 105 (54)

107 inpatient*.tw. (1351)

108 in-patient*.tw. (8268)

109 hospital*.tw. (7143)

110 ward?.tw. (807)

111 medical service.tw. (21)

112 (medical adj2 unit?).tw. (69)

113 surgical service.tw. (5)

114 (surgical adj2 unit?).tw. (63)

115 (H?emodialysis adj2 Unit*).tw. (7)

116 (Dialysis adj2 Unit?).tw. (17)

117 ICU?.tw. (362)

118 intensive care.tw. (1295)

119 (intensive therapy adj2 unit?).tw. (30)

120 (intensive treatment adj2 unit?).tw. (5)

121 (close attention adj2 unit?).tw. (1)

122 (Burn adj2 Unit?).tw. (16)

123 CCU?.tw. (17)

124 (cardiolog* adj2 unit?).tw. (4)

125 (coronary adj2 unit?).tw. (28)

126 coronary care.tw. (28)

127 GICU?.tw. (0)

128 (geriatric adj2 unit?).tw. (11)

129 (gerontolog* adj2 unit?).tw. (0)

130 Recovery Room?.tw. (43)

131 (respiratory care adj2 unit?).tw. (9)

132 (special care adj2 unit?).tw. (99)

133 (nurs* adj2 station?).tw. (7)

134 (nurs* adj2 unit?).tw. (61)

135 operating room?.tw. (138)

136 (self-care adj2 unit?).tw. (0)

137 (Minimal Care adj2 unit?).tw. (0)

138 (high dependency adj2 unit?).tw. (34)

139 (palliative care adj2 unit?).tw. (22)

140 (psychiatr* adj2 unit?).tw. (66)

141 (stroke adj2 unit?).tw. (105)

142 or/107-141 (9336)

143 106 and 142 (53)

Database: Health Technology Assessment <4th Quarter 2016>

Search Strategy:

--------------------------------------------------------------------------------

1 exp clothing/ (9)

2 exp textiles/ (4)

3 1 or 2 (12)

4 exp microcomputers/ (8)

5 exp Telemedicine/ (124)

6 Telemetry/ (17)

7 biosensing techniques/ (4)

8 or/4-7 (143)

9 3 and 8 (0)

10 wearable*.mp. (4)

11 (smart adj5 fabric?).mp. (0)

12 (smart adj5 cloth*).mp. (0)

13 (smart adj5 textile*).mp. (0)

14 (smart adj5 patch??).mp. (0)

15 (smart adj5 glove?).mp. (0)

16 (smart adj5 shoe?).mp. (0)

17 (smart adj5 accessor*).mp. (0)

18 (smart adj5 jewel*).mp. (0)

19 (smart adj5 wrist*).mp. (0)

20 (smart adj5 watch*).mp. (0)

21 smartwatch*.mp. (0)

22 fitbit?.mp. (0)

23 (smart adj5 neckless*).mp. (0)

24 (smart adj5 belt?).mp. (0)

25 (smart adj5 skin).mp. (0)

26 (smart adj5 armband*).mp. (0)

27 (smart adj5 shirt*).mp. (0)

28 (smart adj5 p?jama*).mp. (0)

29 (smart adj5 glasses).mp. (1)

30 (smart adj5 vest?).mp. (0)

31 (smart adj5 garment*).mp. (0)

32 (smart adj5 fashion*).mp. (0)

33 (smart adj5 footwear).mp. (0)

34 (smart adj5 eyewear).mp. (0)

35 (smart adj5 body).mp. (0)

36 (smart adj5 sensor*).mp. (0)

37 (smart adj5 biosensor*).mp. (0)

38 (intelligent adj5 biosensor*).mp. (0)

39 (wireless adj5 biosensor*).mp. (0)

40 (wireless adj2 body).mp. (0)

41 WBAN?.mp. (0)

42 wireless sensor network*.mp. (0)

43 (body adj2 network*).mp. (0)

44 (body adj2 sensor*).mp. (0)

45 personal area network*.mp. (0)

46 (context-aware adj2 system?).mp. (0)

47 (mhealth adj2 patch??).mp. (0)

48 (m-health adj2 patch??).mp. (0)

49 (textile adj3 transducer*).mp. (0)

50 (sensor? adj5 garment*).mp. (0)

51 (biosensor? adj5 garment*).mp. (0)

52 (sensor? adj5 cloth*).mp. (0)

53 (biosensor? adj5 cloth*).mp. (0)

54 (sensor? adj5 fabric*).mp. (0)

55 (biosensor? adj5 fabric*).mp. (0)

56 (bionic adj2 glove*).mp. (0)

57 (high-tech adj3 textile*).mp. (0)

58 (sensor? adj5 textile*).mp. (0)

59 (biosensor? adj5 textile*).mp. (0)

60 (wear* adj3 body).mp. (0)

61 (wear* adj5 device*).mp. (2)

62 (wear* adj5 instrument*).mp. (0)

63 (wear* adj5 smart).mp. (0)

64 (wear* adj5 track*).mp. (0)

65 (wear* adj5 system*).mp. (2)

66 (worn adj3 body).mp. (1)

67 (worn adj5 track*).mp. (0)

68 (worn adj5 smart).mp. (0)

69 (worn adj5 sensor*).mp. (0)

70 (worn adj5 biosensor*).mp. (0)

71 (tech* adj2 tog?).mp. (0)

72 (fashion* adj3 electronic*).mp. (0)

73 (fashion* adj3 tech).mp. (0)

74 (fashion* adj3 technolog*).mp. (0)

75 (connected adj3 cloth*).mp. (0)

76 (bluetooth adj3 cloth*).mp. (0)

77 (internet adj3 cloth*).mp. (0)

78 (digital adj2 cloth*).mp. (0)

79 (advanced adj2 textile*).mp. (0)

80 (electronic* adj2 textile*).mp. (0)

81 e-textile*.mp. (1)

82 (advanced adj2 fabric*).mp. (0)

83 (electronic* adj3 cloth*).mp. (0)

84 (WATCH adj2 society).mp. (0)

85 (life adj2 shirt).mp. (0)

86 (life adj2 vest).mp. (0)

87 body-mount*.mp. (0)

88 (electronic* adj2 skin*).mp. (0)

89 (electronic* adj2 patch??).mp. (0)

90 (activit* adj2 track*).mp. (0)

91 (fitness* adj2 track*).mp. (0)

92 (physical* adj2 track*).mp. (0)

93 or/9-92 (9)

94 limit 93 to english language (8)

Interface - EBSCOhost Research Databases
Database - CINAHL with Full Text

Top of Form

| **#** | **Query** | **Limiters/Expanders** | **Results** |
| --- | --- | --- | --- |
| S169 | S166 OR S168 | Search modes - Boolean/Phrase | 437 |
| S168 | S165 NOT S167 | Search modes - Boolean/Phrase | 421 |
| S167 | S120 AND S164 | Limiters - Age Groups: All Infant, All Child  Search modes - Boolean/Phrase | 42 |
| S166 | S120 AND S164 | Limiters - Age Groups: All Adult  Search modes - Boolean/Phrase | 242 |
| S165 | S120 AND S164 | Search modes - Boolean/Phrase | 463 |
| S164 | S121 OR S122 OR S123 OR S124 OR S125 OR S126 OR S127 OR S128 OR S129 OR S130 OR S131 OR S132 OR S133 OR S134 OR S135 OR S136 OR S137 OR S138 OR S139 OR S140 OR S141 OR S142 OR S143 OR S144 OR S145 OR S146 OR S147 OR S148 OR S149 OR S150 OR S151 OR S152 OR S153 OR S154 OR S155 OR S156 OR S157 OR S158 OR S159 OR S160 OR S161 OR S162 OR S163 | Search modes - Boolean/Phrase | 1,033,219 |
| S163 | TI (stroke N2 unit*) OR AB (stroke N2 unit*) | Search modes - Boolean/Phrase | 1,297 |
| S162 | TI (psychiatr* N2 unit*) OR AB (psychiatr* N2 unit*) | Search modes - Boolean/Phrase | 1,427 |
| S161 | TI (palliative care N2 unit*) OR AB (palliative care N2 unit*) | Search modes - Boolean/Phrase | 1,069 |
| S160 | TI (high dependency N2 unit*) OR AB (high dependency N2 unit*) | Search modes - Boolean/Phrase | 215 |
| S159 | TI (Minimal Care N2 unit*) OR AB (Minimal Care N2 unit*) | Search modes - Boolean/Phrase | 3 |
| S158 | TI (self-care N2 unit*) OR AB (self-care N2 unit*) | Search modes - Boolean/Phrase | 17 |
| S157 | TI GICU* OR AB GICU* | Search modes - Boolean/Phrase | 11 |
| S156 | TI operating room* OR AB operating room* | Search modes - Boolean/Phrase | 4,510 |
| S155 | TI (nurs* N2 unit*) OR AB (nurs* N2 unit*) | Search modes - Boolean/Phrase | 5,702 |
| S154 | TI (nurs* N2 station*) OR AB (nurs* N2 station*) | Search modes - Boolean/Phrase | 216 |
| S153 | TI (special care N2 unit*) OR AB (special care N2 unit*) | Search modes - Boolean/Phrase | 496 |
| S152 | TI (respiratory care N2 unit*) OR AB (respiratory care N2 unit*) | Search modes - Boolean/Phrase | 54 |
| S151 | TI Recovery Room* OR AB Recovery Room* | Search modes - Boolean/Phrase | 382 |
| S150 | TI (gerontolog* N2 unit*) OR AB (gerontolog* N2 unit*) | Search modes - Boolean/Phrase | 16 |
| S149 | TI (geriatric N2 unit*) OR AB (geriatric N2 unit*) | Search modes - Boolean/Phrase | 542 |
| S148 | TI coronary care OR AB coronary care | Search modes - Boolean/Phrase | 825 |
| S147 | TI CCU* OR AB CCU* | Search modes - Boolean/Phrase | 430 |
| S146 | TI (coronary N2 unit*) OR AB (coronary N2 unit*) | Search modes - Boolean/Phrase | 741 |
| S145 | TI (cardiolog* N2 unit*) OR AB (cardiolog* N2 unit*) | Search modes - Boolean/Phrase | 160 |
| S144 | TI (Burn N2 Unit*) OR AB (Burn N2 Unit*) | Search modes - Boolean/Phrase | 1,007 |
| S143 | TI (close attention N2 unit*) OR AB (close attention N2 unit*) | Search modes - Boolean/Phrase | 1 |
| S142 | TI (intensive treatment N2 unit*) OR AB (intensive treatment N2 unit*) | Search modes - Boolean/Phrase | 7 |
| S141 | TI (intensive therapy N2 unit*) OR AB (intensive therapy N2 unit*) | Search modes - Boolean/Phrase | 201 |
| S140 | TI intensive care OR AB intensive care | Search modes - Boolean/Phrase | 32,995 |
| S139 | TI ICU* OR AB ICU* | Search modes - Boolean/Phrase | 14,667 |
| S138 | TI (Dialysis N2 Unit*) OR AB (Dialysis N2 Unit*) | Search modes - Boolean/Phrase | 531 |
| S137 | TI (H#emodialysis N2 Unit*) OR AB (H#emodialysis N2 Unit*) | Search modes - Boolean/Phrase | 454 |
| S136 | TI (surgical N2 unit*) OR AB (surgical N2 unit*) | Search modes - Boolean/Phrase | 2,704 |
| S135 | TI surgical service OR AB surgical service | Search modes - Boolean/Phrase | 403 |
| S134 | TI (medical N2 unit*) OR AB (medical N2 unit*) | Search modes - Boolean/Phrase | 3,308 |
| S133 | TI medical service OR AB medical service | Search modes - Boolean/Phrase | 5,143 |
| S132 | TI medical service | Search modes - Boolean/Phrase | 324 |
| S131 | TI ward* OR AB ward* | Search modes - Boolean/Phrase | 16,420 |
| S130 | TI hospital* OR AB hospital* | Search modes - Boolean/Phrase | 235,687 |
| S129 | TI in-patient* OR AB in-patient* | Search modes - Boolean/Phrase | 819,224 |
| S128 | TI inpatient* OR AB inpatient* | Search modes - Boolean/Phrase | 28,088 |
| S127 | (MH "Patients' Rooms+") | Search modes - Boolean/Phrase | 1,656 |
| S126 | (MH "Hospitals+") | Search modes - Boolean/Phrase | 77,122 |
| S125 | (MH "Hospital Units+") | Search modes - Boolean/Phrase | 62,187 |
| S124 | (MH "Hospitalization+") | Search modes - Boolean/Phrase | 48,047 |
| S123 | (MH "Critically Ill Patients") | Search modes - Boolean/Phrase | 8,679 |
| S122 | (MH "Aged, Hospitalized") | Search modes - Boolean/Phrase | 2,802 |
| S121 | (MH "Inpatients") | Search modes - Boolean/Phrase | 66,774 |
| S120 | S97 AND S119 | Search modes - Boolean/Phrase | 1,749 |
| S119 | S98 OR S99 OR S100 OR S101 OR S102 OR S103 OR S104 OR S105 OR S106 OR S107 OR S108 OR S109 OR S110 OR S111 OR S112 OR S113 OR S114 OR S115 OR S116 OR S117 OR S118 | Search modes - Boolean/Phrase | 138,837 |
| S118 | TI Cardiotocograph* OR AB Cardiotocograph* | Search modes - Boolean/Phrase | 237 |
| S117 | TI tocograph* OR AB tocograph* | Search modes - Boolean/Phrase | 5 |
| S116 | TI neuromonitor* OR AB neuromonitor* | Search modes - Boolean/Phrase | 189 |
| S115 | TI (measure* N3 vital sign*) OR AB (measure* N3 vital sign*) | Search modes - Boolean/Phrase | 291 |
| S114 | TI (track* N3 vital sign*) OR AB (track* N3 vital sign*) | Search modes - Boolean/Phrase | 1 |
| S113 | TI Electrocardiograph* OR AB Electrocardiograph* | Search modes - Boolean/Phrase | 4,085 |
| S112 | TI Capnomet* OR AB Capnomet* | Search modes - Boolean/Phrase | 98 |
| S111 | TI Acceleromet* OR AB Acceleromet* | Search modes - Boolean/Phrase | 3,183 |
| S110 | TI Actigraph* OR AB Actigraph* | Search modes - Boolean/Phrase | 1,227 |
| S109 | TI Polysomnograph* OR AB Polysomnograph* | Search modes - Boolean/Phrase | 1,936 |
| S108 | TI Somnograph* OR AB Somnograph* | Search modes - Boolean/Phrase | 17 |
| S107 | TI (continu* N3 observation*) OR AB (continu* N3 observation*) | Search modes - Boolean/Phrase | 259 |
| S106 | TI (continu* N3 scan*) OR AB (continu* N3 scan*) | Search modes - Boolean/Phrase | 81 |
| S105 | TI (continu* N3 sensing) OR AB (continu* N3 sensing) | Search modes - Boolean/Phrase | 26 |
| S104 | TI (continu* N3 sensor*) OR AB (continu* N3 sensor*) | Search modes - Boolean/Phrase | 166 |
| S103 | TI (continu* N3 detect*) OR AB (continu* N3 detect*) | Search modes - Boolean/Phrase | 217 |
| S102 | TI (continu* N3 record*) OR AB (continu* N3 record*) | Search modes - Boolean/Phrase | 967 |
| S101 | TI (continu* N3 measur*) OR AB (continu* N3 measur*) | Search modes - Boolean/Phrase | 2,340 |
| S100 | TI surveill* OR AB surveill* | Search modes - Boolean/Phrase | 22,954 |
| S99 | TI monitor* OR AB monitor* | Search modes - Boolean/Phrase | 70,733 |
| S98 | (MH "Monitoring, Physiologic+") | Search modes - Boolean/Phrase | 53,631 |
| S97 | S12 OR S13 OR S14 OR S15 OR S16 OR S17 OR S18 OR S19 OR S20 OR S21 OR S22 OR S23 OR S24 OR S25 OR S26 OR S27 OR S28 OR S29 OR S30 OR S31 OR S32 OR S33 OR S34 OR S35 OR S36 OR S37 OR S38 OR S39 OR S40 OR S41 OR S42 OR S43 OR S44 OR S45 OR S46 OR S47 OR S48 OR S49 OR S50 OR S51 OR S52 OR S53 OR S54 OR S55 OR S56 OR S57 OR S58 OR S59 OR S60 OR S61 OR S62 OR S63 OR S64 OR S65 OR S66 OR S67 OR S68 OR S69 OR S70 OR S71 OR S72 OR S73 OR S74 OR S75 OR S76 OR S77 OR S78 OR S79 OR S80 OR S81 OR S82 OR S83 OR S84 OR S85 OR S86 OR S87 OR S88 OR S89 OR S90 OR S91 OR S92 OR S93 OR S94 OR S95 OR S96 | Search modes - Boolean/Phrase | 4,570 |
| S96 | TI (physical* N2 track*) OR AB (physical* N2 track*) | Search modes - Boolean/Phrase | 107 |
| S95 | TI (fitness* N2 track*) OR AB (fitness* N2 track*) | Search modes - Boolean/Phrase | 85 |
| S94 | TI (activit* N2 track*) OR AB (activit* N2 track*) | Search modes - Boolean/Phrase | 250 |
| S93 | TI (electronic* N2 patch*) OR AB (electronic* N2 patch*) | Search modes - Boolean/Phrase | 1 |
| S92 | TI (electronic* N2 skin*) OR AB (electronic* N2 skin*) | Search modes - Boolean/Phrase | 10 |
| S91 | TI body-mount* OR AB body-mount* | Search modes - Boolean/Phrase | 5 |
| S90 | TI (life N2 vest) OR AB (life N2 vest) | Search modes - Boolean/Phrase | 2 |
| S89 | TI (life N2 shirt) OR AB (life N2 shirt) | Search modes - Boolean/Phrase | 1 |
| S88 | TI (WATCH N2 society) OR AB (WATCH N2 society) | Search modes - Boolean/Phrase | 2 |
| S87 | TI (electronic* adj3 cloth*) OR AB (electronic* adj3 cloth*) | Search modes - Boolean/Phrase | 0 |
| S86 | TI (advanced N2 fabric*) OR AB (advanced N2 fabric*) | Search modes - Boolean/Phrase | 5 |
| S85 | TI e-textile* OR AB e-textile* | Search modes - Boolean/Phrase | 1 |
| S84 | TI (electronic* N2 textile*) OR AB (electronic* N2 textile*) | Search modes - Boolean/Phrase | 5 |
| S83 | TI (advanced N2 textile*) OR AB (advanced N2 textile*) | Search modes - Boolean/Phrase | 0 |
| S82 | TI (digital N2 cloth*) OR AB (digital N2 cloth*) | Search modes - Boolean/Phrase | 0 |
| S81 | TI (internet adj3 cloth*) OR AB (internet adj3 cloth*) | Search modes - Boolean/Phrase | 0 |
| S80 | TI (bluetooth adj3 cloth*) OR AB (bluetooth adj3 cloth*) | Search modes - Boolean/Phrase | 0 |
| S79 | TI (connected adj3 cloth*) OR AB (connected adj3 cloth*) | Search modes - Boolean/Phrase | 0 |
| S78 | TI (fashion* adj3 technolog*) OR AB (fashion* adj3 technolog*) | Search modes - Boolean/Phrase | 0 |
| S77 | TI (fashion* adj3 tech) OR AB (fashion* adj3 tech) | Search modes - Boolean/Phrase | 0 |
| S76 | TI (fashion* adj3 electronic*) OR AB (fashion* adj3 electronic*) | Search modes - Boolean/Phrase | 0 |
| S75 | TI (tech* N2 tog*) OR AB (tech* N2 tog*) | Search modes - Boolean/Phrase | 180 |
| S74 | TI (worn N5 biosensor*) OR AB (worn N5 biosensor*) | Search modes - Boolean/Phrase | 1 |
| S73 | TI (worn N5 sensor*) OR AB (worn N5 sensor*) | Search modes - Boolean/Phrase | 55 |
| S72 | TI (worn N5 smart) OR AB (worn N5 smart) | Search modes - Boolean/Phrase | 1 |
| S71 | TI (worn N5 track*) OR AB (worn N5 track*) | Search modes - Boolean/Phrase | 18 |
| S70 | TI (worn adj3 body) OR AB (worn adj3 body) | Search modes - Boolean/Phrase | 0 |
| S69 | TI (wear* N5 system*) OR AB (wear* N5 system*) | Search modes - Boolean/Phrase | 216 |
| S68 | TI (wear* N5 track*) OR AB (wear* N5 track*) | Search modes - Boolean/Phrase | 77 |
| S67 | TI (wear* N5 smart) OR AB (wear* N5 smart) | Search modes - Boolean/Phrase | 14 |
| S66 | TI (wear* N5 instrument*) OR AB (wear* N5 instrument*) | Search modes - Boolean/Phrase | 28 |
| S65 | TI (wear* N5 device*) OR AB (wear* N5 device*) | Search modes - Boolean/Phrase | 464 |
| S64 | TI (wear* adj3 body) OR AB (wear* adj3 body) | Search modes - Boolean/Phrase | 0 |
| S63 | TI (biosensor* N5 textile*) OR AB (biosensor* N5 textile*) | Search modes - Boolean/Phrase | 0 |
| S62 | TI (sensor* N5 textile*) OR AB (sensor* N5 textile*) | Search modes - Boolean/Phrase | 4 |
| S61 | TI (high-tech adj3 textile*) OR AB (high-tech adj3 textile*) | Search modes - Boolean/Phrase | 0 |
| S60 | TI (bionic N2 glove*) OR AB (bionic N2 glove*) | Search modes - Boolean/Phrase | 2 |
| S59 | TI (biosensor* N5 fabric*) OR AB (biosensor* N5 fabric*) | Search modes - Boolean/Phrase | 3 |
| S58 | TI (sensor* N5 fabric*) OR AB (sensor* N5 fabric*) | Search modes - Boolean/Phrase | 12 |
| S57 | TI (biosensor* N5 cloth*) OR AB (biosensor* N5 cloth*) | Search modes - Boolean/Phrase | 0 |
| S56 | TI (sensor* N5 cloth*) OR AB (sensor* N5 cloth*) | Search modes - Boolean/Phrase | 9 |
| S55 | TI (biosensor* N5 garment*) OR AB (biosensor* N5 garment*) | Search modes - Boolean/Phrase | 0 |
| S54 | TI (sensor* N5 garment*) OR AB (sensor* N5 garment*) | Search modes - Boolean/Phrase | 5 |
| S53 | TI (textile adj3 transducer*) OR AB (textile adj3 transducer*) | Search modes - Boolean/Phrase | 0 |
| S52 | TI (m-health N2 patch*) OR AB (m-health N2 patch*) | Search modes - Boolean/Phrase | 0 |
| S51 | TI (mhealth N2 patch*) OR AB (mhealth N2 patch*) | Search modes - Boolean/Phrase | 0 |
| S50 | TI (context-aware N2 system*) OR AB (context-aware N2 system*) | Search modes - Boolean/Phrase | 6 |
| S49 | TI personal area network* OR AB personal area network* | Search modes - Boolean/Phrase | 0 |
| S48 | TI (body N2 sensor*) OR AB (body N2 sensor*) | Search modes - Boolean/Phrase | 120 |
| S47 | TI (body N2 network*) OR AB (body N2 network*) | Search modes - Boolean/Phrase | 38 |
| S46 | TI wireless sensor network* OR AB wireless sensor network* | Search modes - Boolean/Phrase | 2 |
| S45 | TI WBAN* OR AB WBAN* | Search modes - Boolean/Phrase | 1 |
| S44 | TI (wireless N2 body) OR AB (wireless N2 body) | Search modes - Boolean/Phrase | 3 |
| S43 | TI (wireless N5 biosensor*) OR AB (wireless N5 biosensor*) | Search modes - Boolean/Phrase | 1 |
| S42 | TI (intelligent N5 biosensor*) OR AB (intelligent N5 biosensor*) | Search modes - Boolean/Phrase | 0 |
| S41 | TI (smart N5 biosensor*) OR AB (smart N5 biosensor*) | Search modes - Boolean/Phrase | 1 |
| S40 | TI (smart N5 sensor*) OR AB (smart N5 sensor*) | Search modes - Boolean/Phrase | 35 |
| S39 | TI (smart N5 body) OR AB (smart N5 body) | Search modes - Boolean/Phrase | 12 |
| S38 | TI (smart N5 eyewear) OR AB (smart N5 eyewear) | Search modes - Boolean/Phrase | 0 |
| S37 | TI (smart N5 footwear) OR AB (smart N5 footwear) | Search modes - Boolean/Phrase | 1 |
| S36 | TI (smart N5 fashion*) OR AB (smart N5 fashion*) | Search modes - Boolean/Phrase | 2 |
| S35 | TI (smart N5 garment*) OR AB (smart N5 garment*) | Search modes - Boolean/Phrase | 0 |
| S34 | TI (smart N5 vest*) OR AB (smart N5 vest*) | Search modes - Boolean/Phrase | 1 |
| S33 | TI (smart N5 glasses) OR AB (smart N5 glasses) | Search modes - Boolean/Phrase | 8 |
| S32 | TI (smart N5 p?jama) OR AB (smart N5 p?jama) | Search modes - Boolean/Phrase | 0 |
| S31 | TI (smart N5 shirt*) OR AB (smart N5 shirt*) | Search modes - Boolean/Phrase | 4 |
| S30 | TI (smart N5 armband*) OR AB (smart N5 armband*) | Search modes - Boolean/Phrase | 0 |
| S29 | TI (smart N5 skin) OR AB (smart N5 skin) | Search modes - Boolean/Phrase | 11 |
| S28 | TI (smart N5 belt*) OR AB (smart N5 belt*) | Search modes - Boolean/Phrase | 1 |
| S27 | TI (smart N5 neckless*) OR AB (smart N5 neckless*) | Search modes - Boolean/Phrase | 0 |
| S26 | TI fitbit* OR AB fitbit* | Search modes - Boolean/Phrase | 105 |
| S25 | TI smartwatch* OR AB smartwatch* | Search modes - Boolean/Phrase | 29 |
| S24 | TI (smart N5 watch*) OR AB (smart N5 watch*) | Search modes - Boolean/Phrase | 19 |
| S23 | TI (smart N5 wrist*) OR AB (smart N5 wrist*) | Search modes - Boolean/Phrase | 6 |
| S22 | TI (smart N5 jewel*) OR AB (smart N5 jewel*) | Search modes - Boolean/Phrase | 0 |
| S21 | TI (smart N5 accessor*) OR AB (smart N5 accessor*) | Search modes - Boolean/Phrase | 1 |
| S20 | TI (smart N5 shoe*) OR AB (smart N5 shoe*) | Search modes - Boolean/Phrase | 6 |
| S19 | TI (smart N5 glove*) OR AB (smart N5 glove*) | Search modes - Boolean/Phrase | 2 |
| S18 | TI (smart N5 patch*) OR AB (smart N5 patch*) | Search modes - Boolean/Phrase | 3 |
| S17 | TI (smart N5 textile*) OR AB (smart N5 textile*) | Search modes - Boolean/Phrase | 7 |
| S16 | TI (smart N5 cloth*) OR AB (smart N5 cloth*) | Search modes - Boolean/Phrase | 15 |
| S15 | TI (smart N5 fabric*) OR AB (smart N5 fabric*) | Search modes - Boolean/Phrase | 3 |
| S14 | TI wearable* OR AB wearable* | Search modes - Boolean/Phrase | 750 |
| S13 | (MH "Wearable Sensors+") | Search modes - Boolean/Phrase | 2,682 |
| S12 | S4 AND S11 | Search modes - Boolean/Phrase | 71 |
| S11 | S5 OR S6 OR S7 OR S8 OR S9 OR S10 | Search modes - Boolean/Phrase | 18,993 |
| S10 | (MH "Biosensors") | Search modes - Boolean/Phrase | 157 |
| S9 | (MH "Biosensing Techniques+") | Search modes - Boolean/Phrase | 187 |
| S8 | (MH "Telemetry") | Search modes - Boolean/Phrase | 1,099 |
| S7 | (MH "Telehealth+") | Search modes - Boolean/Phrase | 11,028 |
| S6 | (MH "Wireless Communications") | Search modes - Boolean/Phrase | 6,986 |
| S5 | (MH "Wireless Local Area Networks") | Search modes - Boolean/Phrase | 76 |
| S4 | S1 OR S2 OR S3 | Search modes - Boolean/Phrase | 15,741 |
| S3 | (MH "Textiles") | Search modes - Boolean/Phrase | 361 |
| S2 | (MH "Clothing+") | Search modes - Boolean/Phrase | 15,485 |
| S1 | (MH "Fitness Trackers") | Search modes - Boolean/Phrase | 38 |

Bottom of Form
