# Supplementary material for: Evaluating the Validity and Utility of Wearable Technology for Continuously Monitoring Patients in a Hospital Setting: Systematic Review
Source: JMIR Mhealth Uhealth. 2021 Aug 18;9(8):e17411. doi: 10.2196/17411 (PMC8411322; doi:10.2196/17411)
Supplement: Multimedia Appendix 2 [file mhealth_v9i8e17411_app2.docx]

| **Design Requirements** | **Criterion** | **Excellent** | **Good** | **Fair** | **Poor** |
| --- | --- | --- | --- | --- | --- |
| Q1. Was the  percentage of  missing  Wearable/Criterion data given? |  | Percentage of  missing data  described-  number of  participants in  both wearable and  reference  groups included  in the analyses  provided;  relative to total  number of  participants in  the study (i.e. It  is clear that 28  of 30  participants in  the study  provided data  for wearable and  reference  measures) | Percentage  of missing  data NOT  described-  just total  number of  participants  included in  analysis (no  individual  group  numbers), or  no  indication of  how many  including in  the analyses  at all (i.e.  only the  number of  participants  in the study  is reported,  no  indication  how many  were  included in  the analyses  and if any  measures for  either the  wearable or  references  were  missing) |  |  |

| Q2. Was there  a description of  how missing  data were  handled? |  | Described how  missing data  were handled –  describe  explicitly why  data is missing  and how they  dealt with the  missing data  statistically (i.e.  only data with  both wearable and  reference were  included in the  analyses, or all  available data  was included in  the analyses) | Not  described  but it can be  deduced  how missing  items were  handled –  do not state  explicitly  how missing  data was  dealt with,  but can be  deduced  from table  that less  data was  included in  analysis for  each group  than total  number of  participants  in study | Not clear how  missing items were  handled-no  information, for  example table show  missing data but no  explanation |  |
| --- | --- | --- | --- | --- | --- |
| Q3. Was the  sample size  included in the  analysis  adequate?  Assess  1) number of patients as well  2) number of device-reference reading measurement pairs |  | Adequate | Good | Moderate sample size | Small sample |
|  |  | sample size (≥ | sample size | (30-49) | size (<30) |
|  |  | 100) | (50-99) |  |  |
|  |  |  |  |  |  |
|  |  |  |  |  |  |
| Q4. Can the  Criterion(reference) used  or employed be  considered as a  reasonable  ‘gold  standard’? | **Lab:** | Criterion used  can be  considered an  adequate ‘gold  standard’  (evidence  provided) | No evidence  provided,  but  assumable  that the  criterion  used can be  considered  an adequate  ‘gold  standard’  -not  calibrated  treadmill if  applicable | Unclear whether the  criterion used can be  considered an  adequate ‘gold  standard’ | Criterion used  can NOT be  considered an  adequate ‘gold  standard’ (i.e.  self-reported  time in activity  is not a valid  reference  criterion) |
|  | Steps: VO |  |  |  |  |
|  | EE: IC or DC |  |  |  |  |
|  | Sleep: PSG |  |  |  |  |
|  | Distance: laser, |  |  |  |  |
|  | tape measure, |  |  |  |  |
|  | treadmill |  |  |  |  |
|  | Time: |  |  |  |  |
|  | stopwatch(s) |  |  |  |  |
|  |  |  |  |  |  |
|  |  |  |  |  |  |
|  |  |  |  |  |  |
|  |  |  |  |  |  |
|  |  |  |  |  |  |
|  |  |  |  |  |  |

|  | **Free-Living** Accelerometer, except for EE, it should be doubly labelled water or steps could also be pedometer or sleep could be portable monitor |  |  |  |  |
| --- | --- | --- | --- | --- | --- |
| Q5. Were there any important flaws in the design or methods of the study? |  | No other important methodological flaws in the design or execution of the study- wearable need to be on body throughout measurement period, study met all inclusion criteria, and gold standard or adequate reference used |  | Other minor methodological flaws in the design or execution of the study; meets criteria for ‘Excellent’, but (e.g.) no mention of reader having access to raw data or loss to follow up greater than 20% or reference standard NOT adequate or other differences in the way study was executed compared to  similar study | Other important methodological flaws in the design or execution of the study – not meeting inclusion/exclusion criteria under ‘Excellent’ |
| Q6. For continuous scores, did studies report correlations, AUC or BA  plots? |  | Percent difference AND  equivalency OR BA Plot OR SE of means, correlation coefficient, limits of agreement | Percent difference only |  | No PD or way to calculate PD, but has other measures for accuracy (BA plot, SE of means, correlation coefficient or limits of agreement |
